# Senescence-induced endothelial phenotypes underpin immune-mediated senescence surveillance

**DOI:** 10.1101/gad.349585.122

**Published:** 2022-05-01

**Authors:** Kelvin Yin, Daniel Patten, Sarah Gough, Susana de Barros Gonçalves, Adelyne Chan, Ioana Olan, Liam Cassidy, Marta Poblocka, Haoran Zhu, Aaron Lun, Martijn Schuijs, Andrew Young, Celia Martinez-Jimenez, Timotheus Y.F. Halim, Shishir Shetty, Masashi Narita, Matthew Hoare

**Affiliations:** 1 Cancer Research UK Cambridge Institute, University of Cambridge, Cambridge CB2 0RE, United Kingdom;; 2 Helmholtz Pioneer Campus, Helmholtz Zentrum München, 85764 München, Germany;; 3 Institute of Immunology and Immunotherapy, University of Birmingham, Birmingham B15 2TT, United Kingdom;; 4 Tokyo Tech World Research Hub Initiative (WRHI), Institute of Innovative Research, Tokyo Institute of Technology, Yokohama, Kanagawa 226-0026, Japan;; 5 Department of Medicine, University of Cambridge, Cambridge CB2 0QQ, United Kingdom

**Keywords:** senescence, immune surveillance, SASP, endothelium, liver, NF-κB

## Abstract

Senescence is a stress-responsive tumor suppressor mechanism associated with expression of the senescence-associated secretory phenotype (SASP). Through the SASP, senescent cells trigger their own immune-mediated elimination, which if evaded leads to tumorigenesis. Senescent parenchymal cells are separated from circulating immunocytes by the endothelium, which is targeted by microenvironmental signaling. Here we show that SASP induces endothelial cell NF-κB activity and that SASP-induced endothelial expression of the canonical NF-κB component *Rela* underpins senescence surveillance. Using human liver sinusoidal endothelial cells (LSECs), we show that SASP-induced endothelial NF-κB activity regulates a conserved transcriptional program supporting immunocyte recruitment. Furthermore, oncogenic hepatocyte senescence drives murine LSEC NF-κB activity in vivo. Critically, we show two distinct endothelial pathways in senescence surveillance. First, endothelial-specific loss of *Rela* prevents development of Stat1-expressing CD4^+^ T lymphocytes. Second, the SASP up-regulates ICOSLG on LSECs, with the ICOS–ICOSLG axis contributing to senescence cell clearance. Our results show that the endothelium is a nonautonomous SASP target and an organizing center for immune-mediated senescence surveillance.

## Abstract (precis)

In this study, Yin et al. show that SASP induces endothelial cell NF-κB activity and that SASP-induced endothelial expression of the canonical NF-κB component *Rela* underpins senescence surveillance. Their results show that the endothelium is a nonautonomous SASP target and an organizing center for immune-mediated senescence surveillance.

Cellular senescence is an intrinsic tumor suppressor mechanism that leads to an autonomous cell cycle arrest accompanied by development of a complex secretome, termed the senescence-associated secretory phenotype (SASP). The SASP mediates diverse nonautonomous functionalities in the microenvironment and is controlled by transcriptional regulators such as NF-κB (Chien et al. 2011; Lesina et al. 2016), C/EBPβ (Acosta et al. 2008; Kuilman et al. 2008), NOTCH1 (Hoare et al. 2016), and BRD4 (Tasdemir et al. 2016), in addition to post-transcriptional regulation by IL1 (Orjalo et al. 2009), mTOR (Herranz et al. 2015; Laberge et al. 2015), and COX2 (Gonçalves et al. 2021; Wiley et al. 2021). A key activity of SASP signaling is the recruitment of immune cells to eliminate the senescent cell, restoring tissue homeostasis; failure of this senescence surveillance with long-term persistence of senescent cells results in tissue dysfunction (Demaria et al. 2014), aging phenotypes (Ovadya et al. 2018), and tumorigenesis (Kang et al. 2011).

Senescence surveillance has been shown, in different contexts, to be dependent on multiple immune cell subsets (Kale et al. 2020), including CD4^+^ T lymphocytes (Kang et al. 2011), macrophages (Kang et al. 2011; Lujambio et al. 2013; Eggert et al. 2016; Gonçalves et al. 2021), and NK cells (Xue et al. 2007; Ruscetti et al. 2018; Pereira et al. 2019). How the SASP is able to attract, recruit, and activate these immune cells remains unclear. Senescence phenotypes can be transmitted to adjacent cells through SASP-mediated paracrine (Acosta et al. 2013) or direct cell contact (Hoare et al. 2016) routes, providing further complexity to the senescence microenvironment. Inflammatory SASP components (such as IL1, IL6, and IL8) are both targets and activators of transcription factors NF-κB and C/EBPβ. Therefore, “local” inflammatory cytokine signaling from senescent cells can be amplified in a juxtacrine manner (Acosta et al. 2013). However, it remains unclear how senescent cells communicate with more distal circulating immune cells, normally separated from parenchymal cells by the blood vessel endothelium. Our recent study suggested that liver endothelial cells are a nonautonomous target of the SASP (Hoare et al. 2016), where the SASP modulates endothelial-dependent immunocyte recruitment.

Immunocyte recruitment to the liver occurs within the low-flow channels of the hepatic sinusoids, which are lined by liver sinusoidal endothelial cells (LSECs), a unique, specialized endothelial cell forming a physical barrier between the circulation and the liver parenchyma (Shetty et al. 2018). Interactions between LSECs and both resident and circulating immune cells are crucial in recruitment (Shetty et al. 2018) to the liver microenvironment. Under differing conditions, LSECs selectively recruit specific immune subsets with differing functionality, such as T_regs_ and B lymphocytes, through differential expression of adhesion receptors (Shetty et al. 2011, 2012).

The role of LSECs as a major organizing center integrating microenvironmental signaling and controlling liver regeneration has been shown in acute liver injury (Ding et al. 2014). This has prognostic and therapeutic relevance to cancer; treatment-induced senescence (TIS) in mouse models of pancreatic cancer leads to SASP-mediated endothelial activation and the accumulation of CD8^+^ T lymphocytes in the tumors (Ruscetti et al. 2020), leading to potential induced therapeutic vulnerabilities.

We hypothesized that the endothelium could play a key role during immune-mediated senescence surveillance, relaying and amplifying SASP signaling to circulating immune cells, and controlling the infiltration of specific subsets of immunocytes. Through analyses of signaling between senescent and endothelial cells in vitro and in vivo, we describe how the SASP drives NF-κB-dependent endothelial phenotypes, controlling immune activity in the microenvironment and the effectiveness of senescence surveillance.

## Results

### The senescence secretome nonautonomously regulates endothelial behavior

**Figure 1.**
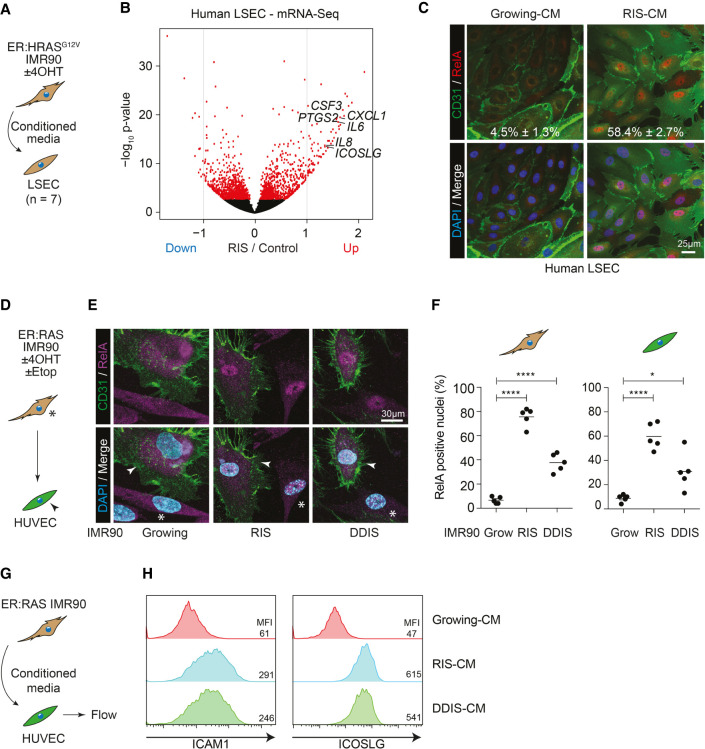
Senescent cells nonautonomously induce NF-κB activity in adjacent endothelial cells. (*A*) Experimental setup: Primary human liver sinusoidal endothelial cells (LSECs) were incubated in conditioned media (CM) from growing or RIS ER:HRAS^G12V^ IMR90 cells for 24 h before harvesting for mRNA sequencing. (*B*) Volcano plot of log_2_ fold change in gene expression against the −log_10_ *P*-value in RIS-CM compared with Grow-CM-treated LSECs. Red dots are significantly differentially expressed (DE; FDR < 0.05) genes. *n* = 7 biological replicates for all conditions. (*C*) Representative immunofluorescence of LSECs from a single donor treated with Grow-CM or RIS-CM and stained for CD31 and RELA. *n* = 3 biological replicates; mean ± SEM. Scale bar, 25 µm. (*D*) Experimental setup: direct coculture of growing, RIS, or DDIS (treated with etoposide) ER:HRAS^G12V^ IMR90 cells (asterisks) with HUVECs (arrowheads). (*E*) Representative immunofluorescence of coculture with senescence-dependent nuclear localization of RELA in both CD31^−^ IMR90s and CD31^+^ HUVECs. Scale bar, 30 µm. (*F*) Separate quantification of RELA nuclear positivity from five biological replicates. Dots are individual replicates, and bars are means. Data were analyzed by one-way ANOVA with Sidak's multiple comparisons test; (*) *P* ≤ 0.05, (****) *P* ≤ 0.0001. (*G*) Experimental setup: HUVECs were incubated in CM from growing, RIS, or DDIS ER:HRAS^G12V^ IMR90 cells for 16 h before flow cytometry. (*H*) Representative flow cytometry histograms of ICAM1 (*left*) and ICOSLG (*right*) expression on HUVECs incubated in the indicated CM. *n* ≥ 3 biological replicates.

Our previous data showed that the SASP from RAS-induced senescent (RIS) IMR90 human diploid fibroblasts (HDFs) could promote lymphocyte adhesion and *trans*-endothelial migration across LSECs (Hoare et al. 2016). This suggested that endothelial cells might be a nonautonomous target of the SASP in the microenvironment, controlling immune cell ingress and thereby senescence surveillance. To understand the molecular basis of induced endothelial phenotypes, we investigated the effects of SASP on the transcriptional profile of LSECs. We obtained primary LSECs from seven patients undergoing liver transplantation for end-stage liver disease (Supplemental Table S1). LSECs were incubated in growing or RIS-conditioned media (CM) (Fig. 1A) from IMR90 cells expressing a 4-hydroxytamoxifen (4-OHT)-inducible form of oncogenic HRAS^G12V^ (ER:HRASG12V) (Hoare et al. 2016) for 24 h before performing mRNA sequencing. This showed RIS-SASP-dependent up-regulation and down-regulation of 555 and 434 genes, respectively (Fig. 1B; Supplemental Table S2), in LSECs. Among genes that were up-regulated by the RIS-SASP across patients were a number of cytokines and chemokines, such as *IL6*, *IL8*, *CXCL1*, and *CSF3*; adhesion molecules such as *ICAM1*; and the immunoregulatory ligand *ICOSLG*. We validated these SASP-induced expression changes at the mRNA level (Supplemental Fig. S1A), and confirmed subsequent protein expression changes through array analyses of CM from SASP-primed LSECs (Supplemental Fig. S1B). This suggests that senescent cells are able to induce a SASP-dependent inflammatory and secretory phenotype in neighboring endothelial cells.

### The SASP nonautonomously regulates NF-κB activity in endothelial cells

To understand SASP-regulated signaling pathways in endothelial cells underpinning this behavior, we conducted transcription factor (TF) motif enrichment analysis on the SASP-regulated genes. The top three predicted TF motifs at the up-regulated genes were binding sites for NF-κB pathway components (Supplemental Fig. S1C). Consistently, gene set enrichment analysis (GSEA) found significant enrichment of endothelial NF-κB target genes (Kempe et al. 2005) in our up-regulated genes in LSECs (Supplemental Fig. S1D). This mirrors potential signaling changes seen in independent transcriptional profiling data from human LSECs during the transition from health to chronic liver disease, where RELA motifs were also highly enriched in genes up-regulated in cirrhosis (Supplemental Fig. S1E; Manicardi et al. 2021).

Activation of the canonical NF-κB pathway leads to the cytoplasmic release and subsequent nuclear translocation of transcriptional regulators, such as RELA. Incubation of LSECs in RIS-CM was associated with an increased nuclear localization of RELA (Fig. 1C). This relocalization of RELA and downstream expression changes were not specific for LSECs, as direct coculture of human umbilical vein endothelial cells (HUVECs) with RIS or DNA damage-induced senescent (DDIS) IMR90s also led to increased nuclear localization of RELA (Fig. 1D–F) and increased expression of IL8 (Supplemental Fig. S1F–H), both autonomously in the IMR90s and nonautonomously in the HUVECs. These induced phenotypes were transmitted through the SASP, as CM from RIS or DDIS, but not from growing IMR90s, led to increased ICAM1 and ICOSLG expression in HUVECs (Fig. 1G,H). The SASP-induced expression changes were similarly broad in HUVECs, as we had shown in LSECs: qPCR analysis showed RIS-CM-induced up-regulation of multiple cytokines and chemokines (Supplemental Fig. S1I).

Previous reports have shown that senescent cells can induce a paracrine senescence in neighboring cells (Acosta et al. 2013). Importantly, in our systems, the SASP drives nonautonomous changes in neighboring endothelial cells without inducing paracrine senescence (Supplemental Fig. S1J–M): There were no changes in *CDKN2A* (the gene encoding p16) or *CCNA2* (Cyclin A2) expression in LSECs after 24 h of incubation in RIS-CM (Supplemental Fig. S1K), or evidence of senescence in HUVECs after 5 d of culture in RIS-CM (Supplemental Fig. S1L,M). Therefore, the SASP from senescent cells induced by different stressors is able to nonautonomously induce canonical NF-κB activation and up-regulation of NF-κB target genes in diverse neighboring endothelial cells without induction of paracrine senescence.

### SASP-induced endothelial NF-κB activity is essential for induced phenotypes

**Figure 2.**
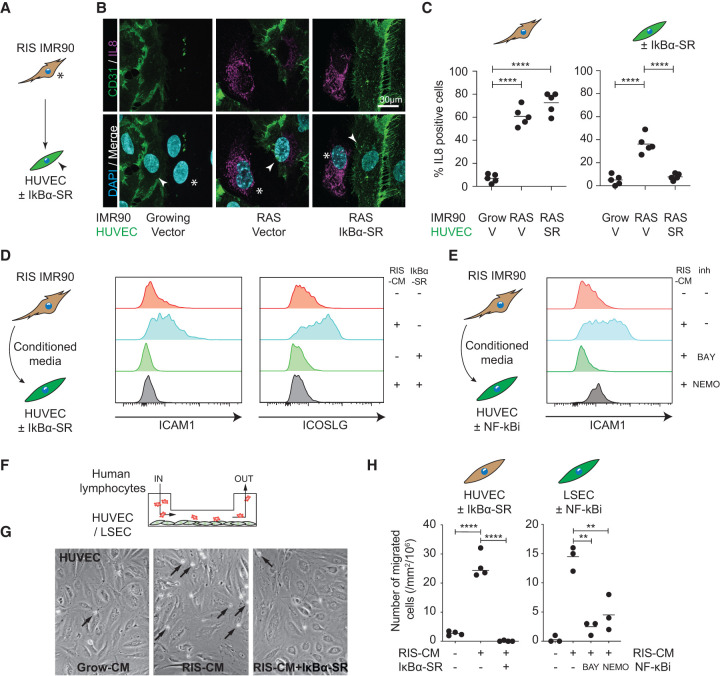
Senescence-induced canonical NF-κB signaling in endothelial cells regulates downstream signaling and lymphocyte recruitment. (*A*) Experimental setup: direct coculture of growing or RIS ER:HRAS^G12V^ IMR90 cells (asterisks) with HUVECs (arrowheads) expressing the IκBα superrepressor (SR) or vector control. (*B*) Representative immunofluorescence of coculture with senescence-dependent IL8 expression in both CD31^−^ IMR90s and CD31^+^ HUVECs. *n* = 5 biological replicates. Scale bar, 30 µm. (*C*) Separate quantification of IL8 positivity from the two cell types. Dots are individual replicates, and bars are means. Data were analyzed by one-way ANOVA with Sidak's multiple comparisons test; (****) *P* ≤ 0.0001. (*D*) Experimental setup: HUVECs expressing the SR or vector control were incubated in CM from growing or RIS ER:HRAS^G12V^ IMR90 cells for 16 h before harvesting for flow cytometry for ICAM1 (*middle* panel) and ICOSLG (*right* panel). *n* ≥ 3 biological replicates. (*E*) Experimental setup: HUVECs were incubated in CM from growing or RIS ER:HRAS^G12V^ IMR90 cells with vehicle or the indicated NF-κB inhibitors for 16 h before harvesting for flow cytometry for ICAM1. *n* ≥ 3 biological replicates. (*F*) Experimental setup: LSECs or HUVECs with the indicated genetic or pharmacological NF-κB inhibitors were incubated in CM from growing or RIS ER:HRAS^G12V^ IMR90 cells for 16 h before performing a flow adhesion assay and analyses of lymphocyte adherence and *trans*-endothelial migration. (*G*) Representative photomicrographs of HUVECs with the indicated constructs and CM, showing adherent lymphocytes (black arrows). (*H*) *Trans*-endothelial migration of lymphocytes in the indicated cell lines and conditions. Dots are individual replicates, and bars are means. Data were analyzed by one-way ANOVA with Sidak's multiple comparisons test; (**) *P* ≤ 0.01, (****) *P* ≤ 0.0001.

To understand the NF-κB dependence of induced endothelial expression changes and functional behaviors, such as lymphocyte capture (Hoare et al. 2016), we ectopically expressed the IκBα^S32A/S36A^ “superrepressor” (SR) in endothelial cells to genetically inhibit NF-κB signaling. Direct coculture of RIS IMR90s with HUVECs led to nuclear localization of RELA (Supplemental Fig. S2A–C) and expression of IL8 (Fig. 2A–C) in both the RIS IMR90s and HUVECs. Ectopic expression of IκBα-SR in HUVECs prevented both the induced nuclear localization of RELA and IL8 expression in the endothelial cells but had no effect on the signal-sending IMR90s. Flow cytometry confirmed that IκBα-SR expression in HUVECs abrogated the SASP-induced up-regulation of ICAM1 and ICOSLG expression (Fig. 2D).

We confirmed these effects using the pharmacological NF-κB inhibitors (NF-κBis) BAY11-7085 (BAY) and the NEMO-binding domain binding peptide (NEMO) during incubation of endothelial cells in RIS-CM. Using ELISA-based pathway activation analyses, incubation of HUVECs in RIS-CM leads to phosphorylation of RELA, but not other intracellular signaling intermediates that might be activated by the RIS-SASP, such as JNK or STAT3 (Supplemental Fig. S2D); both of the NF-κBis prevented RIS-CM-induced phosphorylation of RELA in HUVECs. Treatment of the endothelial cells with NF-κBis abrogated the RIS-CM-induced up-regulation of ICAM1 in HUVECs (Fig. 2E) and ICOSLG in both HUVECs (Supplemental Fig. S2E) and human aortic endothelial cells (HAECs) (Supplemental Fig. S2F), confirming the importance of NF-κB activity in the regulation of these expression changes.

To understand the functional relevance of SASP-induced endothelial NF-κB activity, we used flow adhesion assays (Hoare et al. 2016) to study the ability of peripheral blood lymphocytes (PBLs) from healthy volunteers to adhere to endothelial cells under conditions of shear stress, modeling immune cell recruitment in the liver sinusoids (Fig. 2F). Preincubation of HUVECs in RIS-CM led to an increase in both lymphocyte adhesion (Supplemental Fig. S2G,H) and *trans*-endothelial migration (Fig. 2G,H); both of these activities are completely abrogated when HUVECs express the IκBα-SR. We confirmed that these pathways were preserved in organ-specific endothelium by showing that RIS priming of primary human LSECs led to an increase in both lymphocyte adhesion (Supplemental Fig. S2G,H) and *trans*-endothelial migration; both of these activities were abrogated by treatment with pharmacological NF-κBis (Fig. 2H). Senescence-induced endothelial NF-κB activity in HUVECs is necessary for both adherence and *trans*-endothelial migration of both CD4^+^ and CD8^+^ lymphocytes (Supplemental Fig. S2I,J). Therefore, SASP-mediated nonautonomous induction of endothelial NF-κB activity underpins an inflammatory transcriptional program with cytokine/chemokine secretion and promotes lymphocyte recruitment.

### RIS hepatocytes nonautonomously drive LSEC inflammatory gene expression in vivo

To understand whether senescent cells are able to nonautonomously regulate endothelial cells in vivo, we investigated whether senescent hepatocytes modulate LSEC biology in mice. We use hydrodynamic tail vein (HDTV) injection of transposons containing either oncogenic NRAS^G12V^, leading to mosaic RIS hepatocytes, or a control nonfunctional NRAS^G12V/D38A^ coinjected with a separate plasmid containing the Sleeping Beauty transposase (SB13). This system allowed us to temporally control induction of RIS in a subpopulation of hepatocytes (Kang et al. 2011; Hoare et al. 2016), with senescence established by day 6 after injection and then immune-mediated clearance of the RIS hepatocytes between day 6 and day 12 after injection. To study the effect of RIS hepatocytes on neighboring LSECs, we used endothelial reporter mice: Mice with an inducible endothelial-specific Cre-recombinase (*Cdh5:Cre-ERT2*) and a Cre-activatable LoxP-stop-LoxP-tdTomato reporter were treated with tamoxifen, leading to all endothelial cells, including LSECs, expressing tdTomato. Among nonimmune liver cells, only CD31^+^ endothelial cells express tdTomato after tamoxifen treatment (Supplemental Fig. S3A), suggesting no recombination within hepatocytes. Furthermore, these CD31^+^ cells did not express mVenus after HDTV injection of NRAS^G12V^-IRES-mVenus, suggesting that HDTV injection does not lead to ectopic delivery and expression of NRAS within LSECs.

**Figure 3.**
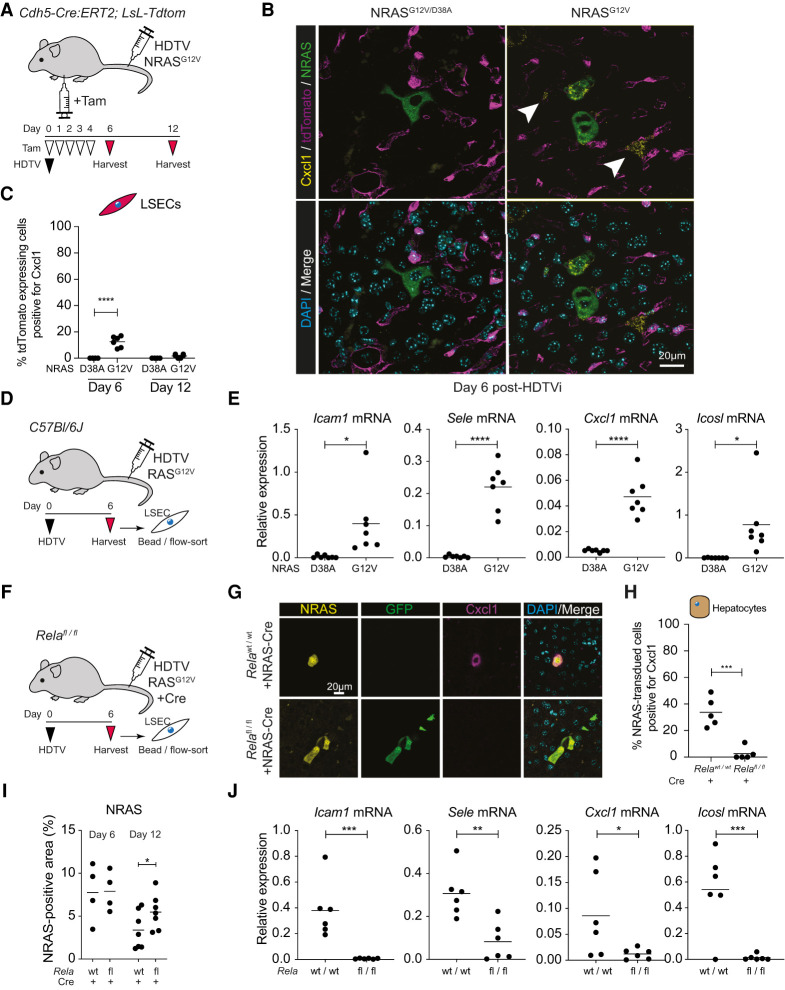
Senescent hepatocytes induce endothelial NF-κB signaling in vivo. (*A*) Experimental setup: Cdh5-Cre:ERT2; LoxP-STOP-LoxP-*TdTomato* endothelial reporter mice underwent hydrodynamic tail vein (HDTV) injection with transposons containing oncogenic NRAS^G12V^ or nonfunctional NRAS^G12V/D38A^ and then intraperitoneal injection with tamoxifen (Tam), leading to TdTomato expression in endothelial cells. (*B*) Representative immunofluorescence of liver sections from mice injected with the indicated constructs, showing Cxcl1 expression in TdTomato^+^ endothelial cells (arrowheads). Scale bar, 20 µm. (*C*) Quantification of Cxcl1-expressing TdTomato^+^ endothelial cells at the indicated time points. Dots are individual mice, and bars are means. Data were analyzed by unpaired Student's *t*-test; (****) *P* ≤ 0.0001. (*D*,*E*) Experimental setup: Wild-type mice were injected with oncogenic NRAS^G12V^ or nonfunctional NRAS^G12V/D38A^ before harvesting and sorting of LSECs at day 6 after HDTV injection (*D*) and qPCR analyses of the indicated gene expression (*E*). Dots are individual mice, and bars are means. Data were analyzed by unpaired Student's *t*-test; (*) *P* ≤ 0.05, (****) *P* ≤ 0.0001. (*F*) Experimental setup: Wild-type or *Rela*^fl/fl^ mice were injected with oncogenic NRAS^G12V^ >> Cre before harvesting at day 6 after HDTV injection. (*G*) Representative immunofluorescence of liver sections for the indicated proteins at day 6 after HDTV injection of NRAS^G12V^ >> Cre-recombinase, leading to RIS with autonomous *Rela* knockout and GFP expression. Scale bar, 20 µm. (*H*) Quantification of Cxcl1-expressing RIS hepatocytes in the indicated conditions. Dots are individual mice, and bars are means. Data were analyzed by unpaired Student's t-test; (***) *P* ≤ 0.001. (*I*) Quantification of NRAS^+^ tissue area in the indicated conditions. Dots are individual mice, and bars are means. Data were analyzed by unpaired Student's *t*-test; (*) *P* ≤ 0.05. (*J*) qPCR analyses of the indicated gene expression from LSECs at day 6 after HDTV injection. Dots are individual mice, and bars are means. Data were analyzed by unpaired Student's *t*-test; (*) *P* ≤ 0.05, (**) *P* ≤ 0.01, (***) *P* ≤ 0.001.

Immunofluorescent staining of livers from these endothelial-reported mice shows that RIS hepatocytes express the SASP component Cxcl1 (Fig. 3B; Supplemental Fig. S3B), as shown previously (Eggert et al. 2016; Gonçalves et al. 2021). However, Cxcl1 expression was also seen in adjacent tdTomato^+^ LSECs at day 6 after HDTV injection with oncogenic NRAS^G12V^, but not after injection of nonfunctional NRAS^G12V/D38A^ (Fig. 3B,C), suggesting that RIS hepatocytes transiently induce Cxcl1 expression in neighboring LSECs. To study whether nonautonomous induction of LSEC genes was more generalized, we induced hepatocyte RIS in wild-type animals before isolating LSECs using a combination of CD146-specific beads and CD31-based flow sorting (Fig. 3D). Compared with control-injected animals, LSECs from mice injected with NRAS^G12V^ have significant up-regulation of *Icam1*, *Sele*, *Cxcl1*, and *Icosl*, the mouse homolog of *ICOSLG* (Fig. 3E). This suggests that RIS hepatocytes nonautonomously induce NF-κB target genes in endothelial cells in vivo that are similar to those we previously identified in human LSECs in vitro (Supplemental Fig. S1A).

Examination of previously published single-cell mRNA sequencing data sets of murine liver endothelial cells (Kalucka et al. 2020) shows a cellular subset that similarly expresses *Sele* and *Icosl* homeostatically (Supplemental Fig. S3C); further examination of the anatomical location of these endothelial cells shows expression of multiple NF-κB target genes, such as *Nfkbia*, *Icam1*, *Sele*, *Cxcl1*, and *Icosl*, in veins and venous capillaries (Supplemental Fig. S3D). Therefore, we have identified a pre-existing endothelial transcriptional program that is nonautonomously induced by neighboring RIS hepatocytes.

Previous studies have shown that the RIS SASP is critically dependent on NF-κB activation (Chien et al. 2011; Lesina et al. 2016). To study whether nonautonomous induction of LSEC gene expression is dependent on the hepatocyte NF-κB activity, we used *Rela*^FL/FL^ mice; upon recombination, *Rela* is deleted, and its transcriptional start site approximated to a downstream *GFP* cassette, leading to GFP expression. HDTV injection of transposons with NRAS^G12V^ and Cre-recombinase outside of the transposon (Gonçalves et al. 2021) led to RIS and autonomous knockout of *Rela* in the same hepatocytes. We confirmed that intrahepatic GFP expression was confined to CD31^−^ cells in the nonimmune compartment, suggesting that Cre-mediated recombination was not occurring in LSECs (Supplemental Fig. S3E). Cxcl1 has previously been shown to be a prominent member of the RIS SASP (Lesina et al. 2016), including hepatocyte SASP (Eggert et al. 2016). Unlike wild-type mice injected with NRAS^G12V^-Cre, *Rela*^FL/FL^ mice failed to express Cxcl1 in RIS hepatocytes (Fig. 3F–H); this was associated with a failure of senescence surveillance with a significant increase in RIS hepatocytes remaining at day 12 after HDTV injection (Fig. 3I; Supplemental Fig. S3F). Analysis of LSECs also showed that there was a failure of nonautonomously driven up-regulation of *Icam1*, *Sele*, *Cxcl1*, and *Icosl* (Fig. 3J). Importantly, in these mice, there were no clear changes in the global abundance of intrahepatic CD4^+^ or CD8^+^ lymphocytes or changes in F4/80-expressing myeloid cells or Klrb1c-expressing NK cells between mice with or without Rela knockout (Supplemental Fig. S3G). All of these immunocytes have been shown to be crucial to senescence surveillance in differing contexts. This shows that RIS hepatocytes require *Rela* to induce SASP gene expression and nonautonomously induce both gene expression in neighboring LSECs and senescence surveillance.

### RIS hepatocytes nonautonomously regulate endothelial NF-κB activity in vivo

**Figure 4.**
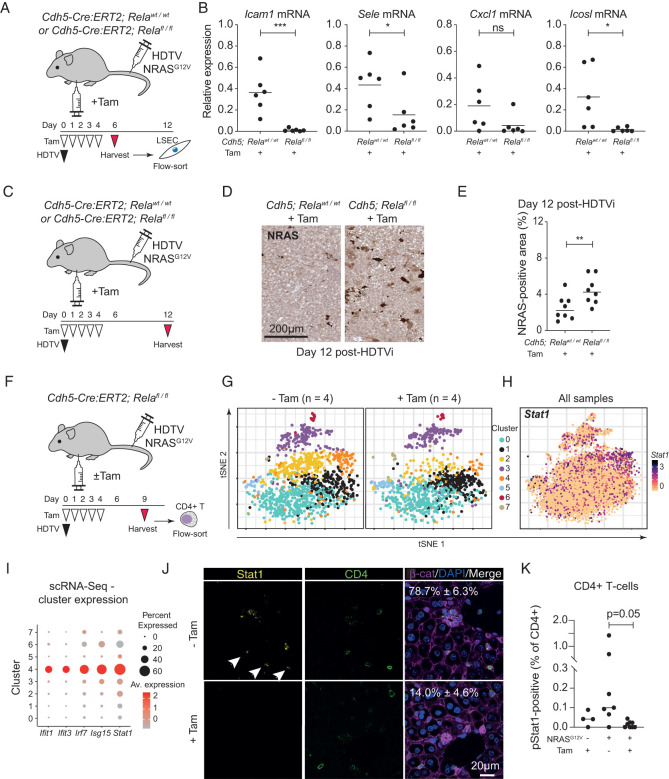
Nonautonomous induction of endothelial NF-κB signaling regulates senescence surveillance and specific immunocyte recruitment. (*A*) Experimental setup: Cdh5-Cre:ERT2; *Rela*^fl/fl^ or *Rela*^wild-type (WT)^ mice underwent hydrodynamic tail vein (HDTV) injection with NRAS^G12V^-containing transposons and then intraperitoneal injection with tamoxifen (Tam), leading to deletion of *Rela* in endothelial cells. (*B*) LSECs were harvested 6 d after HDTV injection and analyzed for the indicated gene expression by qPCR. Dots are individual mice, and bars are means. Data were analyzed by unpaired Student's *t*-test; (*) *P* ≤ 0.05, (***) *P* ≤ 0.001. (*C*) Experimental setup: Cdh5-Cre:ERT2; *Rela*^fl/fl^ or wild-type mice underwent hydrodynamic tail vein (HDTV) injection with transposons containing oncogenic NRAS^G12V^ and then intraperitoneal injection with tamoxifen (Tam) before harvesting at 12 d after HDTV injection and analysis of RIS hepatocytes by immunohistochemistry. (*D*,*E*) Representative photomicrographs of RAS immunochemistry from the indicated conditions (*D*; scale bar, 200 µm) with quantification (*E*). Dots are individual mice, and bars are means. Data were analyzed by unpaired Student's *t*-test; (**) *P* ≤ 0.01. (*F*) Experimental setup: Cdh5-Cre:ERT2; *Rela*^fl/fl^ mice underwent hydrodynamic tail vein (HDTV) injection with NRAS^G12V^-containing transposons and intraperitoneal injection with tamoxifen (Tam) or vehicle control before harvesting at 9 d after HDTV injection. Intrahepatic CD4^+^ T lymphocytes were flow-sorted before droplet-based scRNA sequencing. (*G*,*H*) tSNE clustering of 8152 CD4^+^ T cells from four mice in each indicated condition, showing differential abundance of cells in different clusters (*G*) and log_2_ normalized *Stat1* expression across all eight mice (*H*). (*I*) Expression plot showing cluster-specific expression of the indicated interferon-stimulated genes in the indicated CD4^+^ T-cell clusters from scRNA-seq data. (*J*) Representative immunofluorescence of liver sections from mice (as in *F*) injected with the indicated constructs, showing Stat1 expression in CD4^+^ cells (arrowheads). Scale bar, 20 µm. *n* = 3 biological replicates. (*K*) Flow cytometric analysis of Stat1 phosphorylation at Ser727 in intrahepatic CD4^+^ T lymphocytes in the indicated conditions. Dots are individual mice, and bars are means. Data were analyzed by one-way ANOVA with Sidak's multiple comparisons test.

To understand whether nonautonomous induction of endothelial NF-κB activity is crucial for gene expression and functional changes, we knocked out *Rela* in LSECs during RIS in hepatocytes. We crossed *Cdh5-Cre:ERT2* mice with *Rela*^FL/FL^ mice, allowing tamoxifen-inducible deletion of *Rela* in endothelial cells (Fig. 4A) with demonstrable GFP expression, and therefore Cre-mediated recombination, only within CD31^+^ endothelial cells (Supplemental Fig. S4A).

Although LSECs from wild-type animals undergoing HDTV injection with NRAS^G12V^ showed up-regulation of *Icam1*, *Sele*, *Cxcl1*, and *Icosl* (Fig. 3D), both induced transcriptional changes (Fig. 4A,B) and expression of Cxcl1 protein (Supplemental Fig. S4B) were abrogated when endothelial *Rela* was lost, suggesting that SASP-mediated induction of endothelial NF-κB activity was crucial. Endothelial *Rela* was also crucial for immune-mediated clearance of RIS hepatocytes, as immunohistochemistry showed significant retention of these cells in the livers of knockout animals at day 12 after HDTV injection of NRAS^G12V^ (Fig. 4C–E), suggesting that nonautonomous induction of endothelial NF-κB activity underpins senescence surveillance.

### Senescence-induced endothelial phenotypes control intrahepatic CD4^+^ T lymphocytes in vivo

Previous studies showed that senescence surveillance of murine RIS hepatocytes is dependent on CD4^+^ T lymphocytes (Kang et al. 2011). We had shown that SASP-induced endothelial NF-κB activity was crucial for the recruitment of several T-lymphocyte subsets in vitro, including CD4^+^ T lymphocytes (Supplemental Fig. S2I,J). It remained unclear whether the SASP-primed endothelium simply acts as a gatekeeper to immunocyte ingress or might be an inducible regulator of specific immunocyte functionality. Therefore, we studied the effect of induced endothelial RelA signaling on intrahepatic CD4^+^ T lymphocytes. There were no differences in the global intrahepatic abundance of CD4^+^ cells associated with loss of endothelial *Rela* during induction of hepatocyte RIS (Supplemental Fig. S4C). Therefore, to understand whether induced endothelial NF-κB activity regulates specific CD4^+^ T-cell subsets, we performed multiplexed droplet-based single-cell (sc) RNA sequencing of flow-sorted intrahepatic CD4^+^ T cells at day 9 after HDTV injection with NRAS^G12V^, with or without endothelial-specific deletion of *Rela* (Fig. 4F). This time point represents the period of maximum CD4^+^ T-cell-dependent immune-mediated clearance of RIS hepatocytes (Kang et al. 2011; Hoare et al. 2016; Gonçalves et al. 2021).

Across eight mice, we analyzed 8152 CD4^+^ T cells that fell into eight transcriptional clusters (Fig. 4G), with each cluster containing cells from control and endothelial *Rela* knockout mice. We found that endothelial-specific deletion of *Rela* was associated with a specific loss of CD4^+^ T cells in clusters 2 (lymphotoxin-β^HI^) and 4 during senescence surveillance. Further examination of these two transcriptional subtypes showed that they have the highest expression levels of the transcriptional regulator *Stat1* (Fig. 4H). Stat1 has been shown to be crucial for the development of Th1-polarized effector function in T lymphocytes (Sim et al. 2016) and to be tumor-suppressive in mice (Kaplan et al. 1998). In addition to the changes in *Stat1* expression, cells in cluster 4 also expressed high levels of multiple interferon-stimulated genes (ISGs) such as *Irf7* and *Isg15* (Fig. 4I), suggesting that induced endothelial Rela regulates an interferon-stimulated program in a specific subset of intrahepatic lymphocytes.

### Endothelial *Rela*-dependent activation of Stat1 in CD4^+^ T lymphocytes in vivo

To confirm these changes, we examined the protein expression and activation status of Stat1 in CD4^+^ T lymphocytes in similar mice by immunofluorescence and flow cytometry, respectively. In mice injected with NRAS^G12V^, we identified Stat1-expressing CD4^+^ cells by immunofluorescence. However, when endothelial *Rela* was deleted, there are significantly fewer Stat1-expressing cells (*P* = 0.001) (Fig. 4J). Jak/Stat agonists lead to activating phosphorylation of Stat1 at two C-terminal residues, followed by nuclear translocation and downstream transcriptional regulation. Analysis of phosphorylation of Stat1 at serine 727 (S727) shows that induction of hepatocyte RIS was associated with increased pStat1 in intrahepatic CD4^+^ T lymphocytes (Fig. 4K). Endothelial-specific knockout of *Rela* completely abrogated this Stat1 phosphorylation; analyses of CD8^+^ T lymphocytes from the same mice showed a similar endothelial *Rela*-dependent loss of Stat1 phosphorylation in CD8^+^ T cells (Supplemental Fig. S4D). This suggests that SASP-primed LSECs can regulate specific functionality in intrahepatic T cells through induced NF-κB signaling. Therefore, the SASP-primed endothelium underpins the recruitment and/or activation of specific adaptive immune cell subsets and immune-mediated senescence surveillance.

As our previous data suggest that SASP-primed endothelial cells up-regulate *ICOSLG/Icosl*, we were interested in CD4^+^ T lymphocytes expressing its cognate receptor, *Icos. Icos* expression was localized to cells in clusters 3 (*Cxcr3*^+^ CD4^+^ cells) and 6 (proliferating CD4^+^ cells) (Supplemental Fig. S4E) that express multiple genes suggestive of activation, such as *CD69* and *Ccna2* (Cyclin A2) (Supplemental Fig. S4F). However, upon comparison between NRAS^G12V^-injected control mice and similar mice with endothelial knockout of *Rela*, there was no difference in the relative abundance or transcriptional profile of cells in these clusters, suggesting that there are no clear changes in *Icos*^+^ CD4^+^ T lymphocytes with loss of LSEC RelA-dependent *Icosl* expression.

### Loss of ICOS–ICOSLG signaling impairs senescence surveillance in vivo

**Figure 5.**
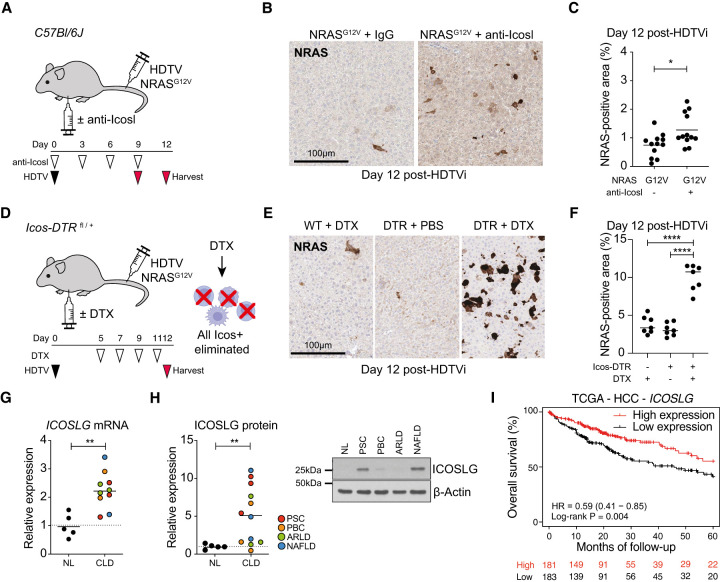
Icos–Icosl signaling regulates senescence surveillance. (*A*) Experimental setup: Wild-type mice were injected with NRAS^G12V^-containing transposons and then received intermittent intraperitoneal injection of anti-Icosl or matched IgG control on the indicated days before harvest on day 9 or 12 after HDTV injection. (*B*,*C*) Representative photomicrographs of RAS immunochemistry from the indicated conditions (*B*; scale bar, 100 µm) with quantification (*C*). Dots are individual mice, and bars are means. Data were analyzed by unpaired Student's *t*-test; (*) *P* ≤ 0.05. (*D*) Experimental setup: *Icos-*DTR mice injected with NRAS^G12V^-containing transposons then received intermittent intraperitoneal injection of diphtheria toxin (DTX) or vehicle control on the indicated days before harvest on day 12 after HDTV injection. (*E*,*F*) Representative photomicrographs of RAS immunochemistry from the indicated conditions (*E*; scale bar, 100 µm) with quantification (*F*). Dots are individual mice, and bars are means. Data were analyzed by one-way ANOVA with Sidak's multiple comparisons test; (****) *P* ≤ 0.0001. (*G*) qRT-PCR analysis of *ICOSLG* expression relative to *ACTB* in samples from normal human livers (*n* = 5) or chronic liver disease of the indicated etiology (*n* = 10). Dots are individual patients, and bars are means. Data were analyzed by unpaired Student's *t*-test; (**) *P* ≤ 0.01. (*H*) Immunoblotting analysis of ICOSLG expression in liver samples from normal human livers (*n* = 5) or chronic liver disease of the indicated etiology (*n* = 12). Dots are individual patients, and bars are means. Data were analyzed by unpaired Student's *t*-test; (**) *P* ≤ 0.01. Example immunoblot of ICOSLG in samples from normal and CLD livers. (*I*) Kaplan–Meier analysis of overall survival for patients with HCC in the TCGA data set dichotomized by *ICOSLG* mRNA expression higher or lower than the median. Data were analyzed by log-rank test.

ICOSLG is an immunoregulatory molecule that, when stimulated by its cognate receptor ICOS, drives T-lymphocyte activation (Dong et al. 2001). Although loss of endothelial RelA did not alter the abundance of intrahepatic *Icos*^+^ CD4^+^ lymphocytes, we hypothesized that SASP-induced *Icosl* expression could modulate other Icos^+^ immunocytes during senescence surveillance. To study whether Icosl was crucial to immune-mediated senescence surveillance in vivo, we studied wild-type mice undergoing HDTV injection with NRAS^G12V^, treated with or without Icosl-blocking antibodies (Fig. 5A). Treatment with Icosl-blocking antibodies led to significantly greater numbers of NRAS-expressing hepatocytes remaining at day 12 after injection (Fig. 5B,C), suggesting that Icosl signaling contributes to immune-mediated senescence surveillance.

As the scRNA-seq data did not show an endothelial-dependent change in *Icos*-expressing intrahepatic CD4^+^ T cells, we elected to study changes in the entire intrahepatic immune system associated with loss of Icosl signaling during senescence surveillance; we studied wild-type mice with or without Icosl blockade at day 9 after injection (Supplemental Fig. S5A,B) using multiplexed mass cytometry (Gonçalves et al. 2021). Using antibodies against 23 cell surface markers, we studied the relative abundance of all immunocyte subsets between conditions (Supplemental Fig. S5C). We confirmed impaired clearance of NRAS-expressing hepatocytes in mice with Icosl blockade (Supplemental Fig. S5A,B). Compared with NRAS^D38A^-injected mice, those injected with NRAS^G12V^ showed significant enrichment in granulocytes and monocytes (Supplemental Fig. S5D), with a reduced abundance of most other immune cell subsets. Upon similar comparison of mice injected with NRAS^G12V^ and Icosl-blocking antibodies with NRAS^G12V^-injected control animals, there was a substantial reversal of all these immune changes, with reduced abundance of granulocytes/monocytes and an increased abundance of all other immunocyte subsets, suggesting that Icosl signaling has broad effects across the immune response.

Expression of the Icos receptor was seen in subsets of CD4^+^ T lymphocytes, macrophages, and Kupffer cells (Supplemental Fig. S5E), the latter expressing high levels of CD68 and F4/80 (Supplemental Fig. S5E). Analysis of independent murine scRNA-seq data (Guilliams et al. 2022) confirms the homeostatic expression of *Icos* in Kupffer cells, in addition to CD4^+^ T lymphocytes (Supplemental Fig. S5F). Therefore, this complete reprogramming of the intrahepatic immune landscape, associated with Icosl blockade, could be a secondary effect through modulation of Icos-expressing CD4^+^ lymphocytes and/or other cells such as Kupffer cells.

To further understand the importance of Icos–Icosl signaling in immune-mediated senescence surveillance, we used *Icos-DTR*^fl/+^ mice in which diphtheria toxin receptor (DTR) is expressed only in cells that also express Icos (Oliphant et al. 2014). Treatment of these mice with diphtheria toxin (DTX) selectively depletes *Icos-*expressing immunocytes (Supplemental Fig. S5G). We induced NRAS^G12V^-mediated hepatocyte senescence before injection of DTX or control (Fig. 5D). Ablation of Icos-expressing cells is associated with retention of NRAS-positive hepatocytes at day 12 after injection (Fig. 5E,F), suggesting that Icos-dependent signaling is crucial to immune-mediated senescence surveillance.

Therefore, SASP-primed endothelial cells are able to regulate the abundance of Stat1-activated CD4^+^ lymphocytes; independently, there was evidence that senescence-induced Icos–Icosl signaling was able to modulate the outcome of immune-mediated senescence surveillance.

### ICOSLG is up-regulated in chronic liver disease and is associated with prognosis

To understand the relevance of ICOSLG up-regulation to human disease, we studied hepatic expression levels in patients with normal livers or chronic liver disease. ICOSLG was significantly up-regulated at both mRNA and protein level in samples from patients with a variety of liver diseases of both metabolic and immune etiologies (Fig. 5G,H). The level of *ICOSLG* expression is also associated with prognosis in patients with HCC from the TCGA data set (Fig. 5I).

## Discussion

The data that we have presented here suggest a paradigm where the SASP from senescent cells modulates the behavior of multiple target cells within the microenvironment, including neighboring endothelial cells. These primed endothelial cells then act as organizing and amplification centers of SASP activity, driving immune-mediated senescence surveillance and clearance of senescent cells. Soluble factors from senescent cells induce LSEC NF-κB activity, crucial for transcriptional and functional changes, including both the recruitment of a specific Stat1-activated subset of CD4^+^ T lymphocytes and the outcome of senescence surveillance (Supplemental Fig. S5H).

This SASP-dependent regulation of endothelial behavior has been shown previously with regulation of neoangiogenesis through IL6 (Ancrile et al. 2007) or VEGF (Coppé et al. 2006) in tumors and ischemic retinopathy (Oubaha et al. 2016). SASP, containing VEGF, was able to drive HUVEC proliferation and invasion but also promoted the vascularization of grafted tumors (Coppé et al. 2006). Recent evidence of senescence priming of the endothelium has emerged: Ruscetti et al. (2020) showed that TIS in murine pancreatic tumors led to vascular remodeling but also CD8^+^ T-lymphocyte recruitment. This T-cell recruitment was dependent on both the SASP and induced endothelial Vcam expression, enhancing sensitivity to immune checkpoint blockade and survival of the mice.

However, the underlying mechanisms of senescence-induced endothelial activation remained unclear. In this present work we have identified induced endothelial NF-κB activation as crucial to senescence surveillance in the liver. However, these induced phenotypes are not persistent: In vitro, there is no evidence that the endothelial cells undergo paracrine senescence; in addition, in vivo, endothelial Cxcl1 expression rapidly wanes with time after induction of hepatocyte senescence. This has been shown before in other contexts: After acute hepatic injury, LSECs mediate liver regeneration through CXCR4 and CXCR7 signaling (Ding et al. 2014). However, during chronic liver injury, LSECs switch to mediate hepatic fibrosis through FGFR1 signaling. Therefore, it may be that the senescence-primed endothelium has a similar switch, only promoting immune cell ingress into the microenvironment during the acute response to senescence, but not during chronic persistent senescence; this remains to be tested. Importantly, this dynamic switch in endothelial phenotype is potentially therapeutically targetable, with promotion of lung and liver regeneration through modulation of induced endothelial phenotypes (Cao et al. 2017).

Downstream from NF-κB, we have identified two pathways of immunocyte modulation that are induced by senescent hepatocytes: the endothelial-dependent induction of Stat1 activation in intrahepatic lymphocytes and the involvement of Icos–Icosl signaling. It has been known for many years that Stat1 is crucial for Ifn-γ expression and cytotoxicity of antitumoral T lymphocytes in vivo (Fallarino and Gajewski 1999); recent work has also shown that loss of inducible STAT1 activation in lymphocytes is a generalized phenomenon in many human cancers and is associated with a failure of lymphocyte activation (Critchley-Thorne et al. 2009). There has been much interest in the role of both inhibitory and stimulatory receptor systems in lymphocyte activation, with clinical deployment of immune checkpoint blockade transforming the prognosis for some cancer patients, including HCC. ICOS–ICOSLG signaling is an activating receptor system (Dong et al. 2001), with ICOS expression only previously described in lymphoid populations. Inducible endothelial ICOSLG expression has been described, particularly after inflammatory stimulation (Khayyamian et al. 2002; Klingenberg et al. 2005), and regulates CD4^+^ (Khayyamian et al. 2002) and CD8^+^ T-lymphocyte functionality during cardiac allograft rejection (Klingenberg et al. 2005), suggesting that inflammatory-induced endothelial ICOSLG expression could activate and shape the local immune reaction at the site of injury or infection. In antitumoral immunity, Icos–Icosl signaling underpins successful antitumoral immune responses after blockade of the inhibitory ligand Ctla4 (Fu et al. 2011) or PD-1 (Hanson et al. 2020), providing rationale for the development of clinical ICOS-agonistic antibodies as second-generation immune oncology agents. These stimulating antibodies seem to avoid the superagonistic activity of earlier CD28 targeted therapies.

Previous studies have shown that elimination of aging- or treatment-induced senescent cells could be beneficial to health span (Baker et al. 2016) and have used directly acting senolytic agents that promote senescent cell apoptosis. Our data suggest rational synergistic treatment targets for senolysis, with potentiation of immune-mediated senescence surveillance through targeting of endothelial pathways with agents such as ICOS-agonistic antibodies. These approaches will require validation in further preclinical models.

## Materials and methods

### Cell culture

IMR90 (ATCC CCL-186; RRID: CVCL_0347) human diploid fibroblasts were cultured in DMEM/10% fetal calf serum (FCS) at 37°C in a 5% O_2_/5% CO_2_ atmosphere. HUVECs (Lonza) and HAECs (Lonza) were cultured in endothelial cell growth medium (Sigma-Aldrich) at 37°C in a 5% O_2_/5% CO_2_ atmosphere. Cell identity was confirmed through short tandem repeat (STR) genotyping, and regular mycoplasma contamination testing was always found to be negative. Cocultures were set up at a cell number ratio of 1:1 and performed in endothelial cell growth medium at 37°C in a 5% O_2_/5% CO_2_ atmosphere. The following drugs and inhibitors were used in cultures: 100 nM 4-hydroxytamoxifen (4OHT) (Sigma), 100 µM etoposide (Etop) (Sigma), 10 µM Bay 11-7085 (Bay11) (Tocris), and NEMO-binding domain binding peptide (NEMO) (Merck Millipore), as indicated in the respective figures.

Conditioned media (CM) was obtained by plating 2.5 × 10^6^ cells in serum-free growth medium for 16 h before filtration through a 0.22-µm pore filter. CM was then supplemented with FCS for a final concentration of 10% FCS-supplemented growth medium, before being applied to HUVECs or LSECs at a ratio of one part CM to three parts fresh endothelial cell growth medium.

### Vectors

The following retroviral vectors were used: pLNCX2 ER:HRAS^G12V^ (Addgene 67844; RRID: Addgene_67844) (Hoare et al. 2016), pBabe-empty vector (Hoare et al. 2016), and pBabe-IκBα^S32A/S36A^-“superrepressor” (a gift from William Hahn; Addgene 15291; RRID: Addgene_15291). The following vectors were used for hydrodynamic tail vein injection: pPGK-SB13 (Kang et al. 2011); pCAGGS-NRAS^G12V^-IRES- (Kang et al. 2011), pCAGGS-NRAS^G12V/D38A^-IRES- (Kang et al. 2011), and pCAGGS-NRAS^G12V^-IRES-mVenus (Hoare et al. 2016); and pCAGGS-NRAS^G12V^-IRES >> Cre (Gonçalves et al. 2021).

### Human LSEC isolation

Tissue and blood samples from patients were obtained with written informed consent and with local ethics committee approval (LREC references 06/Q2702/61, 06/Q2708/61, and 04/Q2708/41). Liver sinusoidal endothelial cells (LSECs) were isolated from human liver tissues, as described previously (Hoare et al. 2016). Briefly, ∼30-g slices of liver tissue were subjected to mechanical chopping with scalpels followed by enzymatic digestion with 10 mg/mL collagenase IA (Sigma-Aldrich). Resultant cell suspensions were layered on a 33%/77% Percoll (GE Healthcare) gradient and centrifuged at 800*g* for 25 min, and the nonparenchymal cell layer was then removed. LSECs were isolated via positive immunomagnetic selection with CD31 Dynabeads (Invitrogen) and resuspended in human endothelial serum-free media (SFM) (Invitrogen) supplemented with 10% human serum (TCS Biosciences), 10 ng/mL vascular endothelial growth factor (VEGF) (PeproTech), and 10 ng/mL hepatocyte growth factor (HGF) (PeproTech). LSECs were seeded on rat tail collagen-coated (1:100; Sigma-Aldrich) culture flasks and maintained in a humidified incubator at 37°C with 5% CO__2__.

### mRNA sequencing of SASP-treated human LSECs

LSECs, obtained from seven explanted livers from patients undergoing liver transplantation (Supplemental Table S1), were incubated in growing CM or RIS-CM from IMR90 cells for 24 h, as described previously (Hoare et al. 2016). RNA was extracted using the Qiagen RNeasy plus kit according to the manufacturer's instructions, and RNA quality was checked using a 4200 Tapestation Bioanalyzer (Agilent). mRNA-seq libraries were prepared as previously described (Hoare et al. 2016), using the Illumina TruSeq stranded mRNA kit and then sequenced on an Illumina HiSeq 2500, obtaining single-end 50-bp reads.

#### Sequencing alignment

mRNA-seq libraries were quality-checked using the FastQC tool from the Babraham Institute. Reads were mapped to the human reference genome hg19 with the STAR (version 2.5.0b) aligner (Harrow et al. 2012), and uniquely mapping reads were selected for further analyses. Read counts were estimated per gene using the featureCounts tool from the subread package against the gene annotation from GENCODE19, using the paired-end and the strand-specific options.

#### Gene set enrichment analyses (GSEAs) and transcription factor motif analyses

Gene set enrichment analyses (GSEAs) and transcription factor motif analyses were performed as described previously (Hoare et al. 2016) using EnrichR against the ENCODE and ChEAconsensus TFs from ChIP-X and the MSigDB Hallmark 2020 databases, which consist of gene sets corresponding to known targets of various transcription factors and pathway members, respectively. The significantly enriched transcription factors and pathways, respectively, were selected using an FDR threshold of 0.05. Enrichment results were plotted using the R package ggplot2 (Wickham 2009). Other data sets used were as follows: Endothelial NF-κB target genes were obtained from the primary publication (Kempe et al. 2005), and regulated LSEC genes in health and chronic liver disease (Manicardi et al. 2021) were obtained from the Gene Expression Omnibus (GSE164799).

### Quantitative reverse transcription PCR

RNA was extracted using the RNeasy Plus kit (Qiagen 74136) according to the manufacturer's instructions and reverse-transcribed to cDNA using the Applied Biosystems high-capacity reverse transcription kit (Thermo Fisher 43-688-13). Relative expression was calculated as previously described (Hoare et al. 2016) on an Applied Biosystems QuantStudio 6 by the 2^−ΔΔCt^ method using β-actin (*ACTB* or *Actb*) as an internal control. The primers used in this study are detailed in Supplemental Table S3.

### Immunofluorescence on cells

Cells were seeded onto #1.5 glass coverslips the day before fixation to achieve 60% confluence. Cells were fixed in 4% (v/v) PFA and permeabilized with 0.2% (v/v) Triton X-100 in PBS. Cells were washed with 0.1% (v/v) Tween in PBS and then blocked with 3% (v/v) serum. Cells were incubated with combinations of the following antibodies: anti-CD31 (1:100; R&D Systems AF806), anti-IL8 (1:100; R&D Systems MAB208), anti-RELA (1:50; Santa Cruz Biotechnology sc-372), anti-RELA (1:400; Cell Signaling 8242), and DAPI (1:1000; Sigma D8417). Coverslips were mounted onto Superfrost Plus slides (Thermo Fisher 4951) with VectaShield antifade mounting medium (Vector Laboratories H-1000).

### Fluorescence microscopy and analysis

Immunohistochemistry slides were scanned on a Leica AT2 at 20× magnification and a resolution of 0.5 µm per pixel. Image analysis was performed using the HALO (Indicalabs) software, applying the Cytonuclear v1.4 algorithm. Positive staining areas were expressed relative (percentage) to the total tissue sectional area (fractional area). Fluorescence images were obtained using a Leica SP5 microscope with a HC PL APO CS2 1.4 NA 63× oil objective (Leica Microsystems). Z-stacks with maximum intensity projection were generated by Fiji/ImageJ software. Fluorescence scanning was performed on a Zeiss Axio Scan.Z1 at 20× magnification and a resolution of 0.5 µm per pixel. Following digitization, image analyses were performed using the Fiji/ImageJ 10.5i software, applying the LoG detector, where blob diameter was set to 0.19 in with a threshold of 0.4 to quantify number of individual nuclei. All images were reviewed manually following analysis to assess accuracy.

### Flow-based adhesion assays

Flow-based adhesion assays (Hoare et al. 2016) were used to study lymphocyte recruitment to LSEC monolayers in vitro under physiological shear stress. Briefly, 7.5 × 10^5^ LSECs were seeded in each channel of a rat tail collagen-coated μ-slide VI 0.4 and grown to confluence overnight. Cells were then stimulated with 1:3 dilution (in whole LSEC media) of growing CM or RIS-CM for 24 h. Peripheral blood lymphocytes (PBLs) were then isolated from whole blood by layering on Lympholyte-H (Cedarlane) and centrifuging at 800*g* for 25 min. The peripheral blood monocytic cell (PBMC) layer was then removed, washed in PBS with 2% FCS and 1 mM EDTA (Gibco by Thermo Fisher Scientific), and centrifuged at 800*g* for 5 min. A platelet depletion step was then performed by a second wash in PBS with 2% FCS and 1 mM EDTA and centrifugation at 350*g* for 10 min. Following this, monocyte depletion via plastic adherence was performed by a 1-h incubation in a cell culture flask. The resultant PBL suspension was washed once again in PBS with 2% FCS and 1 mM EDTA at a cell density of 1 × 10^6^ cells/mL in a flow medium of endothelial SFM with 0.1% BSA. To generate antibody-free purified populations of CD4^+^ and CD8^+^ T lymphocytes, we used a negative bead selection strategy from these PBLs according to the manufacturer's instructions (Dynabeads Untouched CD4^+^/CD8^+^ kits, Thermo Fisher). PBLs or purified subpopulations were then perfused over the LSECs at a physiological shear of 0.05 Pa, with each channel of the μ-slide perfused for 5 min. Afterward, channels were washed thoroughly for 3 min with flow media alone and video recordings were taken. All flow assays were imaged via phase-contrast microscopy on an Olympus IX50 inverted microscope (Olympus), and 12 fields of view from each channel were analyzed for adherent/transmigrated PBLs. Counts of adherent PBLs were then normalized to cells/mm^2^/10^6^ cells perfused using the following equation: {adherent cells/[flow rate (0.28 mL/min)] × bolus (5 min) × field of view area (0.154 mm^2^)} × [1/concentration of lymphocytes (1 × 10^6^ cells/mL)].

### Cytokine array assays

Primary human LSECs were incubated overnight in growing CM or RIS-CM, as above. In the morning, they were washed twice in PBS and incubated in fresh endothelial growth media for 8 h before the LSEC-CM was harvested using the proteome profiler human cytokine array kits (R&D Systems ARY005B) according to the manufacturer's instructions.

### Protein quantification by immunoblotting

Protein was obtained by seeding 2.5 × 10^6^ cells and lysis by RIPA buffer. Immunoblotting was performed, as reported previously (Hoare et al. 2016), using sodium dodecyl sulphate–polyacrylamide gel electrophoresis gels using the following antibodies: anti-β-Actin (Sigma-Aldrich A5441; RRID: AB_476744), anti-ICOSLG (Thermo 16-5889-82; RRID: AB_469129), anti-Cyclin A2 (Sigma c4710; RRID: AB_1078603), anti-HRAS (Calbiochem OP23; RRID: AB_2121030), and anti-p21 (Becton Dikinson 556431; RRID: AB_396415).

### SA-β-galactosidase assays

Growing CM or D6 RIS-CM was harvested from 1.0 × 10^5^ IMR90 cells cultured in serum-free media for 16 h before filtration through a 0.22-μm filter. Growing or RIS IMR90s were subjected to SA-β-gal assay directly. HUVECs were incubated in growing CM or RIS-CM for 5 d before SA-β-gal assays were performed as previously described (Hoare et al. 2016).

### Pathway activation ELISA

HUVECs were incubated overnight in growing CM or RIS-CM with or without NF-κB inhibitors before harvesting of cell lysates and analyses of pathway activation status using the PathScan inflammation multitarget sandwich ELISA kit (Cell Signaling 7276) according to the manufacturer's instructions.

### Mouse models

All animal experiments were approved by the UK Home Office regulations, and mice were group-housed under specific pathogen-free conditions under a 12-h light/dark cycle in accordance with the University of Cambridge guidelines. Mice had free access to water and to standard mouse chow (LabDiet, PicoLab rodent diet 20). Mice genotypes from ear clipping biopsies were determined by Transnetyx (Transnetyx).

Wild-type C57Bl/6, *Rela^fl/fl^* (B6.129S1-Rela^tm1Ukl^/J; MGI: 6148061), and *Rosa26_LoxP-STOP-LoxP-*Tdtomato [Ai9; B6.Cg-*Gt(ROSA)26Sor^tm9(CAG-tdTomato)Hze^*/J; MGI: 3809523] mice were purchased from Charles River. To specifically achieve Cre-mediated recombination in the endothelium, the *Cdh5-Cre:ERT2* [Tg(Cdh5-cre/ERT2)^1Rha^; MGI: 3848982] mouse line that expresses tamoxifen-inducible Cre-recombinase (Cre:ERT2) under the regulation of the vascular endothelial cadherin promoter (VE-Cad; a kind gift from Professor Neil Henderson University of Edinburgh, UK) was used. The *Cdh5-Cre:ERT2;Rela^fl/fl^* mouse strain was generated by crossing *Cdh5-Cre:ERT2* mice with *Rela^fl/fl^* mice. The *Cdh5-Cre:ERT2;Rosa26_LSL-Tdtomato* mouse strain was generated by crossing *Cdh5-Cre:ERT2* mice with *Rosa26_LSL-Tdtomato* mice. To induce recombination, 100 µL of tamoxifen in corn oil solution (20 mg/mL) was given via intraperitoneal injection once every 24 h for five consecutive days.

For in vivo Icosl blockade experiments, C57Bl/6 mice received HDTV injection on day 0, before intraperitoneal injection of InVivoMab rat IgG2a isotype (Bio X Cell BE0089, RRID:AB_1107769) or InVivoMab antimouse Icosl (CD275) (Bio X Cell BE0028, RRID:AB_1107566) on days 0, 3, 6, and 9 at a concentration of 100 µg per animal per injection.

For Icos-DTR experiments, Icos-DTR mice (Oliphant et al. 2014) carrying a floxed DTx receptor (DTR) gene in the *Icos* locus, leading to a null allele (*Icos*^tm1.1(Hbegf)Anjm^; MGI: 6256735; a kind gift from Dr. Andrew McKenzie, Cambridge), received HDTV injection on day 0 before receiving intraperitoneal DTx (25 ng/g of body weight) on days 5, 7, 9, and 11. The efficiency of depletion of Icos^+^ cells was checked by flow cytometry of CD45^+^ splenocytes.

### Hydrodynamic tail vein (HDTV) injection

Mice receiving the HDTV procedure were injected at 5–8 wk of age, as previously described (Hoare et al. 2016). Vectors for injection were prepared with the Qiagen EndoFree MaxiPrep kit. Briefly, 20 µg of the appropriate vector and 5 µg of SB13 transposase-containing plasmid were diluted in sterile physiological saline to a total volume of 10% of the mouse body weight before being injected into the lateral tail vein in <10 sec.

### Immunohistochemistry and immunofluorescence on tissues

Tissues were fixed in 10% neutral-buffered formalin solution for 24 h and transferred to 70% ethanol. Tissues were embedded in paraffin, cut in 3-µm sections on poly-lysine-coated slides, deparaffinized, and rehydrated. Antigen retrieval was performed by incubating slides in either citrate (pH 6) or Tris-EDTA (pH 9) solutions and heat-inducing them in a pressure cooker for 5 min at 120°C. Slides were blocked with donkey serum (Sigma Aldrich D9663) and mouse-on-mouse blocking reagent (Vector Laboratories MKB-2213-1) and pretreated with Vector TrueView autofluorescence quenching kit (Vector Laboratories). All antibodies were prepared in 0.1% (v/v) Tween in PBS containing 300 mM NaCl. Sections were incubated with primary antibodies and prepared as detailed in Supplemental Table S3 before visualization using the DAKO Envision kit according to the manufacturer's instructions and counterstaining with hematoxylin or staining with the appropriate fluorescent-tagged secondary antibodies. Sections were then stained with DAPI (Sigma) before being mounted with VectaShield Vibrance antifade mounting medium.

### Murine LSEC isolation

Murine livers were obtained and dissociated using the liver dissociation kit (Miltenyi Biotec) in accordance with the manufacturer's instructions. Dissociated hepatic cells were passed through a 40-µm filter, and red blood cells (RBCs) were lysed by RBC lysis buffer (eBioscience). The cell suspension was washed with PEB buffer (0.5% [v/v] BSA, 5 mM EDTA in PBS) before incubating in CD146 MicroBeads (Miltenyi Biotec) for 15 min at 4°C. LSECs were enriched by passing the cell suspension through the MACS separator unit (Miltenyi Biotec). The enriched LSEC suspension was washed several times with PEB buffer and stained with Fc receptor blocker (antimouse FCGR3/CD16^+^-FCGR2B/CD32; Biolegend 101301, RRID:AB_312800). Subsequently, cells were stained with anti-CD31-PE (1:50; Thermo Fisher Scientific 12-0311-82, RRID:AB_465632) for 30 min at 4°C and then stained with DAPI (1:1000; Sigma D8417). Cells were sorted using a FACS Aria or Influx (BD Bioscience) directly into RLT plus buffer (Qiagen), passed through a QIAshredder (Qiagen), and stored at −80°C until use.

### Murine hepatic immune cell isolation

Murine livers were obtained and dissociated using the liver dissociation kit (Miltenyi Biotec) in accordance with the manufacturer's instructions. Dissociated hepatic cells were passed through a 40-µm filter, and red blood cells (RBCs) were lysed by RBC lysis buffer (eBioscience). The cell suspension was washed with PEB buffer (0.5% [v/v] BSA, 5 mM EDTA in PBS). The immune cells were enriched from the cell suspension by gradient centrifugation using Optiprep density gradient medium (Sigma-Aldrich). Briefly, the cells were suspended in 10 mL of 33.3% Optiprep diluted in PEB. Seven milliliters of PEB was carefully layered on top of the cell suspension without disturbing the interface of two layers. The cell suspension was centrifuged at 500*g* for 20 min at 4°C without brake at the end of centrifugation. Immune cells at the interface between two layers were extracted, washed with PEB, and analyzed by flow cytometry. Cells were also fixed and stored at −80°C for mass cytometry.

### Flow cytometry

Cultured cells were dislodged from culture vessels nonenzymatically with Versene (Thermo Fisher) in order to maintain cell surface antigens. All cell suspensions were incubated in Fc receptor blocker (Biolegend, as above) in FACS buffer (3% [v/v] BSA in PBS) prior to staining in FACS buffer with fluorochrome-conjugated antibodies (Supplemental Table S3) and an amine-reactive liver/dead dye. For analyses of Stat1 phosphorylation, cells were fixed and permeabilized using the FoxP3/transcription factor fix/perm kit (Invitrogen). Counting beads were added to each sample before processing on the flow cytometer set up with appropriate compensation using single-stained controls. All samples were analyzed using a FACS LSR II, Fortessa, or Symphony (BD Biosciences). Data were further analyzed using FlowJo v10.

### Single-cell sequencing of murine hepatic CD4^+^ T cells

#### Sample preparation, library preparation, and sequencing

Eight *Cdh5;Rela^fl/fl^* mice were used. On day 0, all mice were subjected to HDTV injection of NRAS^G12V^, as previously described. The mice received 100 µL of 20 mg/mL tamoxifen or corn oil solution via intraperitoneal injection once every 24 h for a total of five consecutive days (day 0–4). On day 9 after HDTV injection, all mice were sacrificed and livers were harvested and dissociated using the liver dissociation kit in accordance with the manufacturer's protocol (Miltenyi Biotec). A small lobe of liver was reserved for paraffin embedding. Isolation of murine hepatic immune population was performed as previously described. Importantly, CD4^+^ T-cell purification kit (Miltenyi Biotec 130-104-454) was used to enrich an untouched CD4^+^ T population. One million purified CD4^+^ T cells from each liver were collected and individually labeled with hashtag oligos according to the manufacturer's protocol (Biolegend, TotalSeq-B v3.1 single index) (hashtag oligo labeling sequences are listed in Supplemental Table S3). Following labeling, the samples were washed and pooled before staining with an antibody cocktail containing anti-CD3-PE (1:100; Biolegend 17A2), anti-CD4-BV605 (1:100; Biolegend RM4-5), and fixable viability dye eFluor 780 (1:100; eBioscience 65-0865-14). Stained cells were washed and sorted using an Influx (BD Bioscience) to obtain live ultrapure CD3^+^CD4^+^ T cells.

A total of 50,000 cells was loaded per lane on a 10x Chromium Genomics platform, with the expectancy rate of capture at 60%. Standard 10x Genomics parameters were used as follows: read1: 28 bp, index1: 8 bp, index2: 0 bp, and read2: 91 bp. Downstream single-cell sequencing library preparation followed the manufacturer's instructions (10x Genomics Chromium single-cell 3′ GEM v3 workflow). The library was then sequenced on two lanes of an SP flow cell on an Illumina NovaSeq6000, providing 979 million reads in total.

#### Preprocessing, quality control, and analyses

Raw sequencing reads were mapped to the GRCm38, assembly version 93 (version STAR-2.7.1a). Demultiplexing of hashtag oligos was performed to assign individual cells to respective hashtag barcodes by implementing the HTODemux function of Seurat 3.0 (Satija Laboratory). Quality control was applied by imposing a threshold of <10% reads mapped to mitochondrial genes and removing non-T-cell clusters (mainly hepatocytes expressing *Alb, Apoe*, and *Ttr*). Dimensionality reduction followed by clustering of the data set was obtained by using modularity-based Louvain clustering algorithm by running the Seurat functions FindNeighbours and FindClusters, setting resolution parameter to 0.3. Tsne plots for visualization were produced by the RunTSNE function of Seurat on the basis of principal components. Key marker genes from CD4^+^ T-cell lineages (Supplemental Table S3) were used for cluster annotation.

### Mass cytometry

Murine immune cells were prepared according to the manufacturer's guidance (Fluidigm), and mass cytometry was performed as described previously (Gonçalves et al. 2021). Briefly, immune cells were stained with 5-µm cisplatin in PBS for 5 min at room temperature and then washed with Maxpar staining buffer (MSB). Resuspended cells were fixed in fix I buffer for 10 min at room temperature. The cell suspensions were washed four times in MSB and stored at −80°C as cell pallets in 1-million-cell aliquots. Cell pallets were thawed on ice on the day of use. Each cell pallet was resuspended in Maxpar barcode perm buffer (Fluidigm) and barcoded using the palladium isotope barcodes for 30 min at room temperature. Each barcoded sample was washed twice with MSB before combining into one single-cell suspension, before staining with Fc-Block (BD Bioscience) followed by staining with a mixture of metal-conjugated antibodies directed against extracellular antigens for 30 min at room temperature. In some cases, fluorophore-conjugated antibodies were used as primary antibody before recognition by a metal-conjugated secondary antibody. Titration for each antibody is detailed in Supplemental Table S3. After staining, the cell pallet was washed with MSB and then incubated in Cell-ID Intercalator-Ir overnight at 4°C. The cell pellet was washed four times with MSB and submitted for data acquisition on a Helios CyTOF (Fluidigm). Following acquisition, times series were normalized to internal bead standards, concatenated, and debarcoded using inbuilt software (Fluidigm). Mass cytometry analyses were performed as previously described (Lun et al. 2017).

### Statistics and reproducibility

Statistical analyses were conducted using GraphPad Prism 8 and R statistical software, except where indicated. Statistical details of the experiments are in the relevant figure legends, including the statistical tests used and the number of biological replicates. Unless otherwise stated, data are represented by the mean ± SEM. *n* values represent the number of independent experiments performed or the number of individual mice per condition. One-way ANOVA with Tukey's or Sidak's correction for multiple comparisons was used for data sets with more than two conditions. Student's *t*-tests were used for two-condition comparisons. The statistical tests were justified as appropriate based on the number of samples compared and the assumed variance within populations. A *P-*value of <0.05 was used to indicate statistical significance.

### Data availability

The mRNA sequencing data generated for this study have been deposited at the Gene Expression Omnibus (GEO) in superseries GSE195616. The mRNA sequencing data from CM-treated human LSECs are available at accession number GSE171147, and the scRNA sequencing data from intrahepatic CD4^+^ T lymphocytes in endothelial *Rela* knockout mice are available at GSE195615.

## Competing interest statement

S.d.B.G. is currently an employee of Astra Zeneca. The other authors declare no competing interests.

## Supplementary Material

Supplemental Material

## References

[GAD349585YINC1] Acosta JC, O'Loghlen A, Banito A, Guijarro MV, Augert A, Raguz S, Fumagalli M, Da Costa M, Brown C, Popov N, 2008. Chemokine signaling via the CXCR2 receptor reinforces senescence. Cell 133: 1006–1018. 10.1016/j.cell.2008.03.038 18555777

[GAD349585YINC2] Acosta JC, Banito A, Wuestefeld T, Georgilis A, Janich P, Morton JP, Athineos D, Kang T-W, Lasitschka F, Andrulis M, 2013. A complex secretory program orchestrated by the inflammasome controls paracrine senescence. Nat Cell Biol 15: 978–990. 10.1038/ncb2784 23770676 PMC3732483

[GAD349585YINC3] Ancrile B, Lim K-H, Counter CM. 2007. Oncogenic Ras-induced secretion of IL6 is required for tumorigenesis. Genes Dev 21: 1714–1719. 10.1101/gad.1549407 17639077 PMC1920165

[GAD349585YINC4] Baker DJ, Childs BG, Durik M, Wijers ME, Sieben CJ, Zhong J, Saltness RA, Jeganathan KB, Verzosa GC, Pezeshki A, 2016. Naturally occurring p16Ink4a-positive cells shorten healthy lifespan. Nature 530: 184–189. 10.1038/nature16932 26840489 PMC4845101

[GAD349585YINC5] Cao Z, Ye T, Sun Y, Ji G, Shido K, Chen Y, Luo L, Na F, Li X, Huang Z, 2017. Targeting the vascular and perivascular niches as a regenerative therapy for lung and liver fibrosis. Sci Transl Med 9: eaai8710. 10.1126/scitranslmed.aai8710 28855398 PMC5606244

[GAD349585YINC6] Chien Y, Scuoppo C, Wang X, Fang X, Balgley B, Bolden JE, Premsrirut P, Luo W, Chicas A, Lee CS, 2011. Control of the senescence-associated secretory phenotype by NF-κB promotes senescence and enhances chemosensitivity. Gene Dev 25: 2125–2136. 10.1101/gad.17276711 21979375 PMC3205583

[GAD349585YINC7] Coppé J-P, Kauser K, Campisi J, Beauséjour CM. 2006. Secretion of vascular endothelial growth factor by primary human fibroblasts at senescence. J Biol Chem 281: 29568–29574. 10.1074/jbc.M603307200 16880208

[GAD349585YINC8] Critchley-Thorne RJ, Simons DL, Yan N, Miyahira AK, Dirbas FM, Johnson DL, Swetter SM, Carlson RW, Fisher GA, Koong A, 2009. Impaired interferon signaling is a common immune defect in human cancer. Proc National Acad Sci 106: 9010–9015. 10.1073/pnas.0901329106 PMC2690021 19451644

[GAD349585YINC9] Demaria M, Ohtani N, Youssef SA, Rodier F, Toussaint W, Mitchell JR, Laberge R-M, Vijg J, Van Steeg H, Dollé MET, 2014. An essential role for senescent cells in optimal wound healing through secretion of PDGF-AA. Dev Cell 31: 722–733. 10.1016/j.devcel.2014.11.012 25499914 PMC4349629

[GAD349585YINC10] Ding B-S, Cao Z, Lis R, Nolan DJ, Guo P, Simons M, Penfold ME, Shido K, Rabbany SY, Rafii S. 2014. Divergent angiocrine signals from vascular niche balance liver regeneration and fibrosis. Nature 505: 97–102. 10.1038/nature12681 24256728 PMC4142699

[GAD349585YINC11] Dong C, Juedes AE, Temann UA, Shresta S, Allison JP, Ruddle NH, Flavell RA. 2001. ICOS co-stimulatory receptor is essential for T-cell activation and function. Nature 409: 97–101. 10.1038/35051100 11343121

[GAD349585YINC12] Eggert T, Wolter K, Ji J, Ma C, Yevsa T, Klotz S, Medina-Echeverz J, Longerich T, Forgues M, Reisinger F, 2016. Distinct functions of senescence-associated immune responses in liver tumor surveillance and tumor progression. Cancer Cell 30: 533–547. 10.1016/j.ccell.2016.09.003 27728804 PMC7789819

[GAD349585YINC13] Fallarino F, Gajewski TF. 1999. Cutting edge: differentiation of antitumor CTL in vivo requires host expression of Stat1. J Immunol Baltim Md 1950 163: 4109–4113. 10510345

[GAD349585YINC14] Fu T, He Q, Sharma P. 2011. The ICOS/ICOSL pathway is required for optimal antitumor responses mediated by anti-CTLA-4 therapy. Cancer Res 71: 5445–5454. 10.1158/0008-5472.CAN-11-1138 21708958

[GAD349585YINC15] Gonçalves S, Yin K, Ito Y, Chan A, Olan I, Gough S, Cassidy L, Serrao E, Smith S, Young A, 2021. COX2 regulates senescence secretome composition and senescence surveillance through PGE2. Cell Rep 34: 108860. 10.1016/j.celrep.2021.108860 33730589 PMC7972992

[GAD349585YINC16] Guilliams M, Bonnardel J, Haest B, Vanderborght B, Wagner C, Remmerie A, Bujko A, Martens L, Thoné T, Browaeys R, 2022. Spatial proteogenomics reveals distinct and evolutionarily conserved hepatic macrophage niches. Cell 185: 379–396.e38. 10.1016/j.cell.2021.12.018 35021063 PMC8809252

[GAD349585YINC17] Hanson A, Elpek K, Duong E, Shallberg L, Fan M, Johnson C, Wallace M, Mabry GR, Sazinsky S, Pepper L, 2020. ICOS agonism by JTX-2011 (vopratelimab) requires initial T cell priming and Fc cross-linking for optimal T cell activation and anti-tumor immunity in preclinical models. PLoS One 15: e0239595. 10.1371/journal.pone.0239595 32970735 PMC7514066

[GAD349585YINC18] Harrow J, Frankish A, Gonzalez JM, Tapanari E, Diekhans M, Kokocinski F, Aken BL, Barrell D, Zadissa A, Searle S, 2012. GENCODE: the reference human genome annotation for the ENCODE project. Genome Res 22: 1760–1774. 10.1101/gr.135350.111 22955987 PMC3431492

[GAD349585YINC19] Herranz N, Gallage S, Mellone M, Wuestefeld T, Klotz S, Hanley CJ, Raguz S, Acosta JC, Innes AJ, Banito A, 2015. mTOR regulates MAPKAPK2 translation to control the senescence-associated secretory phenotype. Nat Cell Biol 17: 1205–1217. 10.1038/ncb3225 26280535 PMC4589897

[GAD349585YINC20] Hoare M, Ito Y, Kang T-W, Weekes MP, Matheson NJ, Patten DA, Shetty S, Parry AJ, Menon S, Salama R, 2016. NOTCH1 mediates a switch between two distinct secretomes during senescence. Nat Cell Biol 18: 979–992. 10.1038/ncb3397 27525720 PMC5008465

[GAD349585YINC21] Kale A, Sharma A, Stolzing A, Desprez P-Y, Campisi J. 2020. Role of immune cells in the removal of deleterious senescent cells. Immun Ageing 17: 16. 10.1186/s12979-020-00187-9 32518575 PMC7271494

[GAD349585YINC22] Kalucka J, de Rooij LPMH, Goveia J, Rohlenova K, Dumas SJ, Meta E, Conchinha NV, Taverna F, Teuwen L-A, Veys K, 2020. Single-cell transcriptome atlas of murine endothelial cells. Cell 180: 764–779.e20. 10.1016/j.cell.2020.01.015 32059779

[GAD349585YINC23] Kang T-W, Yevsa T, Woller N, Hoenicke L, Wuestefeld T, Dauch D, Hohmeyer A, Gereke M, Rudalska R, Potapova A, 2011. Senescence surveillance of pre-malignant hepatocytes limits liver cancer development. Nature 479: 547–551. 10.1038/nature10599 22080947

[GAD349585YINC24] Kaplan DH, Shankaran V, Dighe AS, Stockert E, Aguet M, Old LJ, Schreiber RD. 1998. Demonstration of an interferon γ-dependent tumor surveillance system in immunocompetent mice. Proc National Acad Sci 95: 7556–7561. 10.1073/pnas.95.13.7556 PMC22681 9636188

[GAD349585YINC25] Kempe S, Kestler H, Lasar A, Wirth T. 2005. NF-κB controls the global pro-inflammatory response in endothelial cells: evidence for the regulation of a pro-atherogenic program. Nucleic Acids Res 33: 5308–5319. 10.1093/nar/gki836 16177180 PMC1226313

[GAD349585YINC26] Khayyamian S, Hutloff A, Büchner K, Gräfe M, Henn V, Kroczek RA, Mages HW. 2002. ICOS-ligand, expressed on human endothelial cells, costimulates Th1 and Th2 cytokine secretion by memory CD4^+^ T cells. Proc Natl Acad Sci 99: 6198–6203. 10.1073/pnas.092576699 11983910 PMC122926

[GAD349585YINC27] Klingenberg R, Autschbach F, Gleissner C, Giese T, Wambsganss N, Sommer N, Richter G, Katus HA, Dengler TJ. 2005. Endothelial inducible costimulator ligand expression is increased during human cardiac allograft rejection and regulates endothelial cell-dependent allo-activation of CD8^+^ T cells in vitro. Eur J Immunol 35: 1712–1721. 10.1002/eji.200425727 15864782

[GAD349585YINC28] Kuilman T, Michaloglou C, Vredeveld LCW, Douma S, van Doorn R, Desmet CJ, Aarden LA, Mooi WJ, Peeper DS. 2008. Oncogene-induced senescence relayed by an interleukin-dependent inflammatory network. Cell 133: 1019–1031. 10.1016/j.cell.2008.03.039 18555778

[GAD349585YINC29] Laberge R-M, Sun Y, Orjalo AV, Patil CK, Freund A, Zhou L, Curran SC, Davalos AR, Wilson-Edell KA, Liu S, 2015. MTOR regulates the pro-tumorigenic senescence-associated secretory phenotype by promoting IL1A translation. Nat Cell Biol 17: 1049–1061. 10.1038/ncb3195 26147250 PMC4691706

[GAD349585YINC30] Lesina M, Wörmann SM, Morton J, Diakopoulos KN, Korneeva O, Wimmer M, Einwächter H, Sperveslage J, Demir IE, Kehl T, 2016. Rela regulates CXCL1/CXCR2-dependent oncogene-induced senescence in murine Kras-driven pancreatic carcinogenesis. J Clin Invest 126: 2919–2932. 10.1172/JCI86477 27454298 PMC4966329

[GAD349585YINC31] Lujambio A, Akkari L, Simon J, Grace D, Tschaharganeh DF, Bolden JE, Zhao Z, Thapar V, Joyce JA, Krizhanovsky V, 2013. Non-cell-autonomous tumor suppression by p53. Cell 153: 449–460. 10.1016/j.cell.2013.03.020 23562644 PMC3702034

[GAD349585YINC32] Lun ATL, Richard AC, Marioni JC. 2017. Testing for differential abundance in mass cytometry data. Nat Methods 14: 707–709. 10.1038/nmeth.4295 28504682 PMC6155493

[GAD349585YINC33] Manicardi N, Fernández-Iglesias A, Abad-Jordà L, Royo F, Azkargorta M, Ortega-Ribera M, Sanfeliu-Redondo D, Martínez-Alcocer A, Elortza F, Hessheimer AJ, 2021. Transcriptomic profiling of the liver sinusoidal endothelium during cirrhosis reveals stage-specific secretory signature. Cancers (Basel) 13: 2688. 10.3390/cancers13112688 34072510 PMC8198220

[GAD349585YINC34] Oliphant CJ, Hwang YY, Walker JA, Salimi M, Wong SH, Brewer JM, Englezakis A, Barlow JL, Hams E, Scanlon ST, 2014. MHCII-mediated dialog between group 2 innate lymphoid cells and CD4^+^ T cells potentiates type 2 immunity and promotes parasitic Helminth expulsion. Immunity 41: 283–295. 10.1016/j.immuni.2014.06.016 25088770 PMC4148706

[GAD349585YINC35] Orjalo AV, Bhaumik D, Gengler BK, Scott GK, Campisi J. 2009. Cell surface-bound IL-1α is an upstream regulator of the senescence-associated IL-6/IL-8 cytokine network. Proc National Acad Sci 106: 17031–17036. 10.1073/pnas.0905299106 PMC2761322 19805069

[GAD349585YINC36] Oubaha M, Miloudi K, Dejda A, Guber V, Mawambo G, Germain M-A, Bourdel G, Popovic N, Rezende FA, Kaufman RJ, 2016. Senescence-associated secretory phenotype contributes to pathological angiogenesis in retinopathy. Sci Transl Med 8: 362ra144. 10.1126/scitranslmed.aaf9440 27797960

[GAD349585YINC37] Ovadya Y, Landsberger T, Leins H, Vadai E, Gal H, Biran A, Yosef R, Sagiv A, Agrawal A, Shapira A, 2018. Impaired immune surveillance accelerates accumulation of senescent cells and aging. Nat Commun 9: 5435. 10.1038/s41467-018-07825-3 30575733 PMC6303397

[GAD349585YINC38] Pereira BI, Devine OP, Vukmanovic-Stejic M, Chambers ES, Subramanian P, Patel N, Virasami A, Sebire NJ, Kinsler V, Valdovinos A, 2019. Senescent cells evade immune clearance via HLA-E-mediated NK and CD8^+^ T cell inhibition. Nat Commun 10: 2387. 10.1038/s41467-019-10335-5 31160572 PMC6547655

[GAD349585YINC39] Ruscetti M, Leibold J, Bott MJ, Fennell M, Kulick A, Salgado NR, Chen C-C, Ho Y, Sanchez-Rivera FJ, Feucht J, 2018. NK cell–mediated cytotoxicity contributes to tumor control by a cytostatic drug combination. Science 362: 1416–1422. 10.1126/science.aas9090 30573629 PMC6711172

[GAD349585YINC40] Ruscetti M, Morris JP, Mezzadra R, Russell J, Leibold J, Romesser PB, Simon J, Kulick A, Ho Y, Fennell M, 2020. Senescence-induced vascular remodeling creates therapeutic vulnerabilities in pancreas cancer. Cell 181: 424–441.e21. 10.1016/j.cell.2020.03.008 32234521 PMC7278897

[GAD349585YINC41] Shetty S, Weston CJ, Oo YH, Westerlund N, Stamataki Z, Youster J, Hubscher SG, Salmi M, Jalkanen S, Lalor PF, 2011. Common lymphatic endothelial and vascular endothelial receptor-1 mediates the transmigration of regulatory T cells across human hepatic sinusoidal endothelium. J Immunol 186: 4147–4155. 10.4049/jimmunol.1002961 21368224 PMC6016742

[GAD349585YINC42] Shetty S, Bruns T, Weston CJ, Stamataki Z, Oo YH, Long HM, Reynolds GM, Pratt G, Moss P, Jalkanen S, 2012. Recruitment mechanisms of primary and malignant B cells to the human liver. Hepatology 56: 1521–1531. 10.1002/hep.25790 22508288

[GAD349585YINC43] Shetty S, Lalor PF, Adams DH. 2018. Liver sinusoidal endothelial cells—gatekeepers of hepatic immunity. Nat Rev Gastroentero 15: 555–567. 10.1038/s41575-018-0020-y PMC7096836 29844586

[GAD349585YINC44] Sim GC, Wu S, Jin L, Hwu P, Radvanyi LG. 2016. Defective STAT1 activation associated with impaired IFN-γ production in NK and T lymphocytes from metastatic melanoma patients treated with IL-2. Oncotarget 7: 36074–36091. 10.18632/oncotarget.8683 27153543 PMC5094984

[GAD349585YINC45] Tasdemir N, Banito A, Roe J-S, Alonso-Curbelo D, Camiolo M, Tschaharganeh DF, Huang C-H, Aksoy O, Bolden JE, Chen C-C, 2016. BRD4 connects enhancer remodeling to senescence immune surveillance. Cancer Discov 6: 612–629. 10.1158/2159-8290.CD-16-0217 27099234 PMC4893996

[GAD349585YINC46] Wickham H. 2009. ggplot2: elegant graphics for data analysis. Springer Science and Business Media, New York.

[GAD349585YINC47] Wiley CD, Sharma R, Davis SS, Lopez-Dominguez JA, Mitchell KP, Wiley S, Alimirah F, Kim DE, Payne T, Rosko A, 2021. Oxylipin biosynthesis reinforces cellular senescence and allows detection of senolysis. Cell Metab 33: 1124–1136.e5. doi: 10.1016/j.cmet.2021.03.008 33811820 PMC8501892

[GAD349585YINC48] Xue W, Zender L, Miething C, Dickins RA, Hernando E, Krizhanovsky V, Cordon-Cardo C, Lowe SW. 2007. Senescence and tumour clearance is triggered by p53 restoration in murine liver carcinomas. Nature 445: 656–660. 10.1038/nature05529 17251933 PMC4601097

