# Supplementary material for: Senescence-induced endothelial phenotypes underpin immune-mediated senescence surveillance
Source: Genes Dev. 2022 May 1;36(9-10):533–49. doi: 10.1101/gad.349585.122 (PMC9186388; doi:10.1101/gad.349585.122)
Supplement: Supplemental Material [file supp_36_9-10_533__DC1.html]

Senescence-induced endothelial phenotypes underpin immune-mediated senescence surveillance — Supplemental Material 

# Senescence-induced endothelial phenotypes underpin immune-mediated senescence surveillance

## Supplemental Material

- Supp\_FigureS1.ai
- Supp\_FigureS2.ai
- Supp\_FigureS3.ai
- Supp\_FigureS4.ai
- Supp\_FigureS5.ai
- Supp\_Table\_1.xlsx
- Supp\_Table\_2.xlsx
- Supp\_Table\_3.xlsx
