# Supplementary material for: Senescence-induced endothelial phenotypes underpin immune-mediated senescence surveillance
Source: Genes Dev. 2022 May 1;36(9-10):533–49. doi: 10.1101/gad.349585.122 (PMC9186388; doi:10.1101/gad.349585.122)
Supplement: Supplemental Material [file supp_gad.349585.122_Supp_FigureS1.ps]

GENESDEV/2022/349585, Supplementary Figure S1, Yin et al.

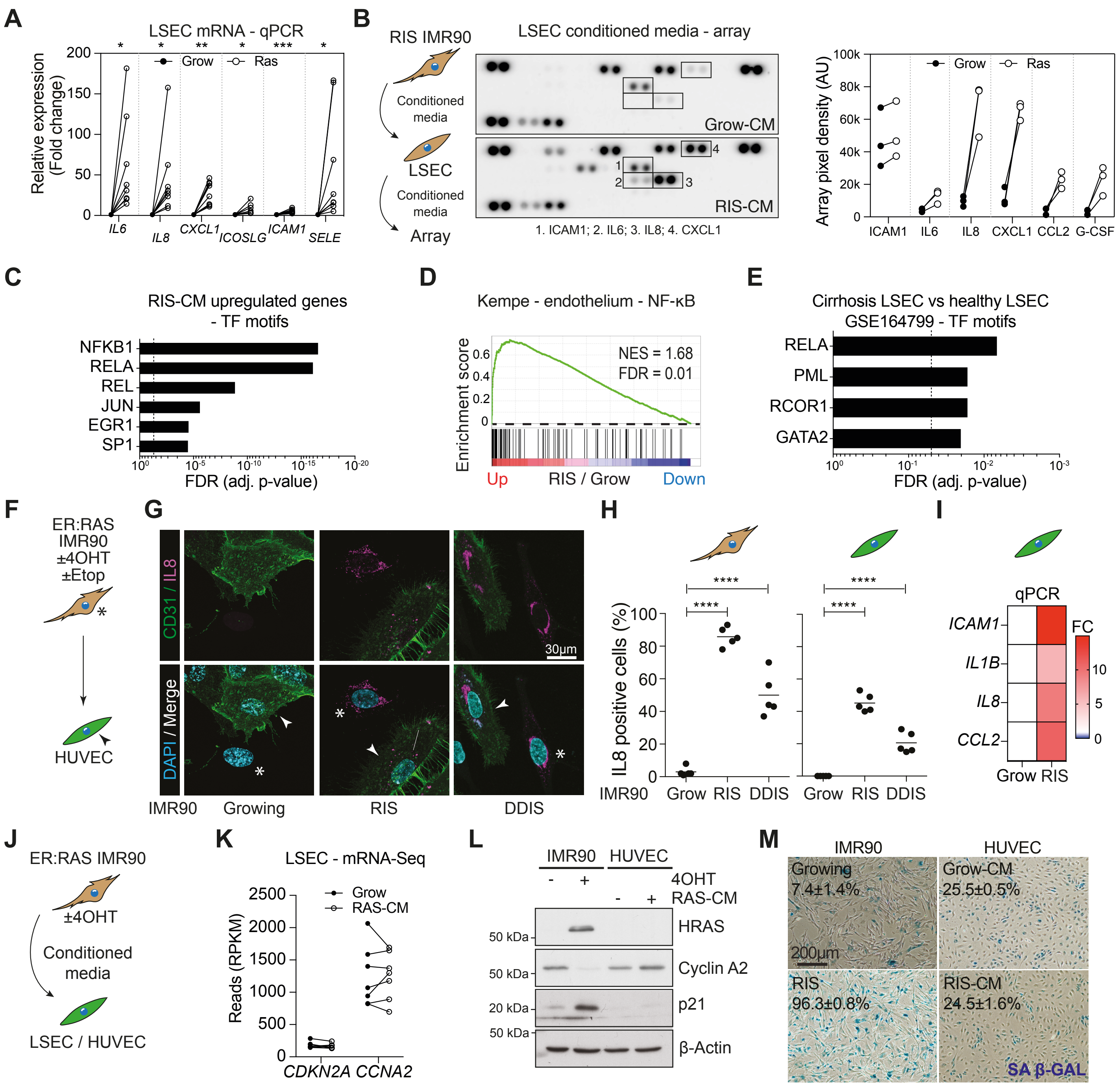

Supplementary figure S1. Senescent cells non-autonomously induce NF-κB activity in adjacent endothelial cells. (A) qPCR analyses of indicated gene expression in human LSECs incubated in growing or RIS-CM (as in Figure 1A); n ≥ 8 biological replicates; dots and lines are individual patients; data analysed by paired student's t-test; *P ≤ 0.05, **P ≤ 0.01, ***P ≤ 0.001. (B) Cytokine array analysis of the secretome of LSECs after they have been incubated in growing or RIS-CM; left, experimental setup; middle, example image from array from Grow-CM (upper) or RIS-CM (lower) with specific proteins indicated; right, quantification of protein dot intensity from n = 3 biological replicates for indicated proteins. (C) Transcription factor motif enrichment analysis for RIS-CM regulated genes in human LSECs from mRNA-Seq data (as in figure 1A-B). (D) Geneset-enrichment analysis (GSEA) demonstrating significant enrichment of previously identified endothelial NF-κB target-genes 27 in our RIS-CM regulated genes. (E) Transcription factor motif enrichment analysis for differentially-regulated genes in LSECs in the context of cirrhosis compared to LSECs in healthy liver (Manicardi et al). (F) Experimental setup: direct co-culture of growing, RIS or DDIS (treated with etoposide) ER:HRAS^{G12V} IMR90 cells (stars) with HUVECs (arrow-heads). (G) Representative immunofluorescence of co-culture with senescence-dependent expression of IL8 in both CD31- IMR90s and CD31+ HUVECs (scale bar 30µm). (H) Separate quantification of IL8 expression from 5 biological replicates; dots are individual replicates; bars are means; data analysed by 1-way ANOVA with Sidak's multiple comparisons test; ****P ≤ 0.0001. (I) Heatmap of qPCR analysis of indicated gene expression from grow- or RIS-CM treated HUVECs; results expressed as fold-change (FC) relative to grow-CM treated condition; n = 4 biological replicates. (J) Experimental setup: LSECs / HUVECs were incubated in CM from growing or RIS ER:HRAS^{G12V} IMR90 cells for 24 hours and 5 days respectively, before separate analyses of markers of senescence by mRNA expression by mRNA-Sequencing of LSECs (K), indicated protein expression by immunoblotting (L) or SA β-Gal expression (M) in HUVECs. (M) Example photomicrographs of indicated cells for SA
