# Supplementary material for: Senescence-induced endothelial phenotypes underpin immune-mediated senescence surveillance
Source: Genes Dev. 2022 May 1;36(9-10):533–49. doi: 10.1101/gad.349585.122 (PMC9186388; doi:10.1101/gad.349585.122)
Supplement: Supplemental Material [file supp_gad.349585.122_Supp_FigureS2.ps]

GENESDEV/2022/349585, Supplementary Figure S2, Yin et al.

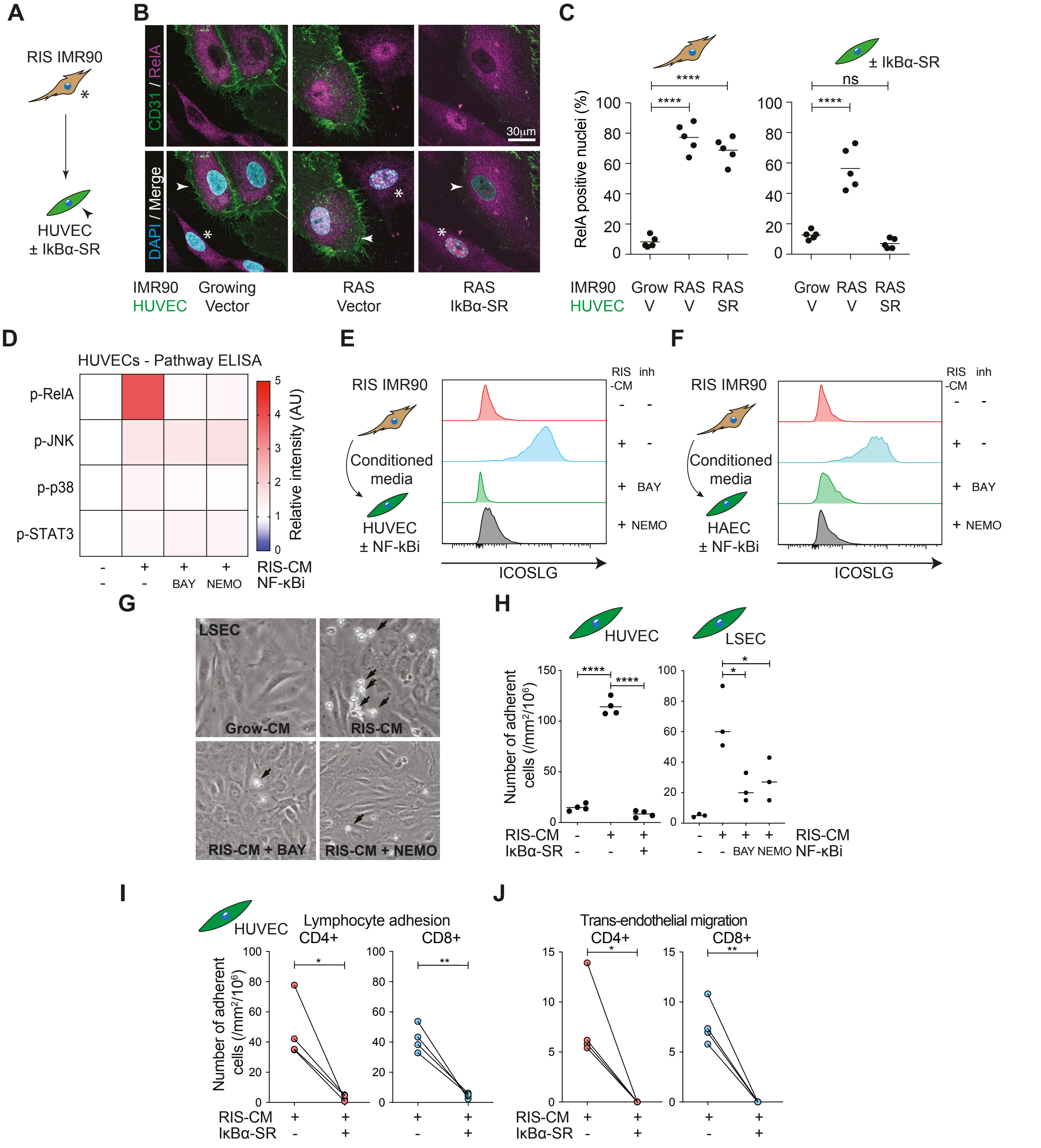

Supplementary figure S2. Senescence-induced canonical NF-κB signalling in endothelial cells regulates downstream signalling and lymphocyte recruitment. (A) Experimental setup: direct co-culture of growing or RIS ER:HRAS^{G12V} IMR90 cells (stars) with HUVECs (arrow-heads) expressing the IκBα-super repressor (SR) or vector control. (B) Representative immunofluorescence of co-culture with senescence-dependent nuclear localisation of RELA in both CD31- IMR90s and CD31+ HUVECs; n = 5 biological replicates (scale bar 30µm). (C) Separate quantification of RELA nuclear positivity from the two cell types; dots are individual replicates; bars are means; data analysed by 1-way ANOVA with Sidak's multiple comparisons test; ****P ≤ 0.0001. (D) Heatmap of intracellular pathway activation analyses using pathway ELISA from the indicated conditions and cells; results expressed relative to grow-CM treated condition; n = 2 biological replicates. (E, F) Experimental setup: HUVECs (E) or human aortic endothelial cells (HAEC, F) were incubated in CM from growing or RIS ER:HRAS^{G12V} IMR90 cells with vehicle or the indicated NF-κB inhibitors for 16 hours before harvesting for flow cytometry for ICOSLG; n ≥ 3 biological replicates. (G) Representative photomicrographs of LSECs with the NF-κB inhibitors for 16 hours and then CM, showing adherent lymphocytes under flow conditions (black arrows). (H) Adherence of lymphocytes in the indicated cell lines and conditions; dots are individual replicates; bars are means; data analysed by 1-way ANOVA with Sidak's multiple comparisons test; ** P ≤ 0.01, ****P ≤ 0.0001. (I) Specific analyses of adherence of sorted CD4+ and CD8+ T-lymphocytes under flow conditions in the indicated cell lines and conditions; dots are individual replicates; bars are means; data analysed by paired student's t-test; *P ≤ 0.05, ** P ≤ 0.01.
