# Supplementary material for: Senescence-induced endothelial phenotypes underpin immune-mediated senescence surveillance
Source: Genes Dev. 2022 May 1;36(9-10):533–49. doi: 10.1101/gad.349585.122 (PMC9186388; doi:10.1101/gad.349585.122)
Supplement: Supplemental Material [file supp_gad.349585.122_Supp_FigureS3.ps]

GENESDEV/2022/349585, Supplementary Figure S3, Yin et al.

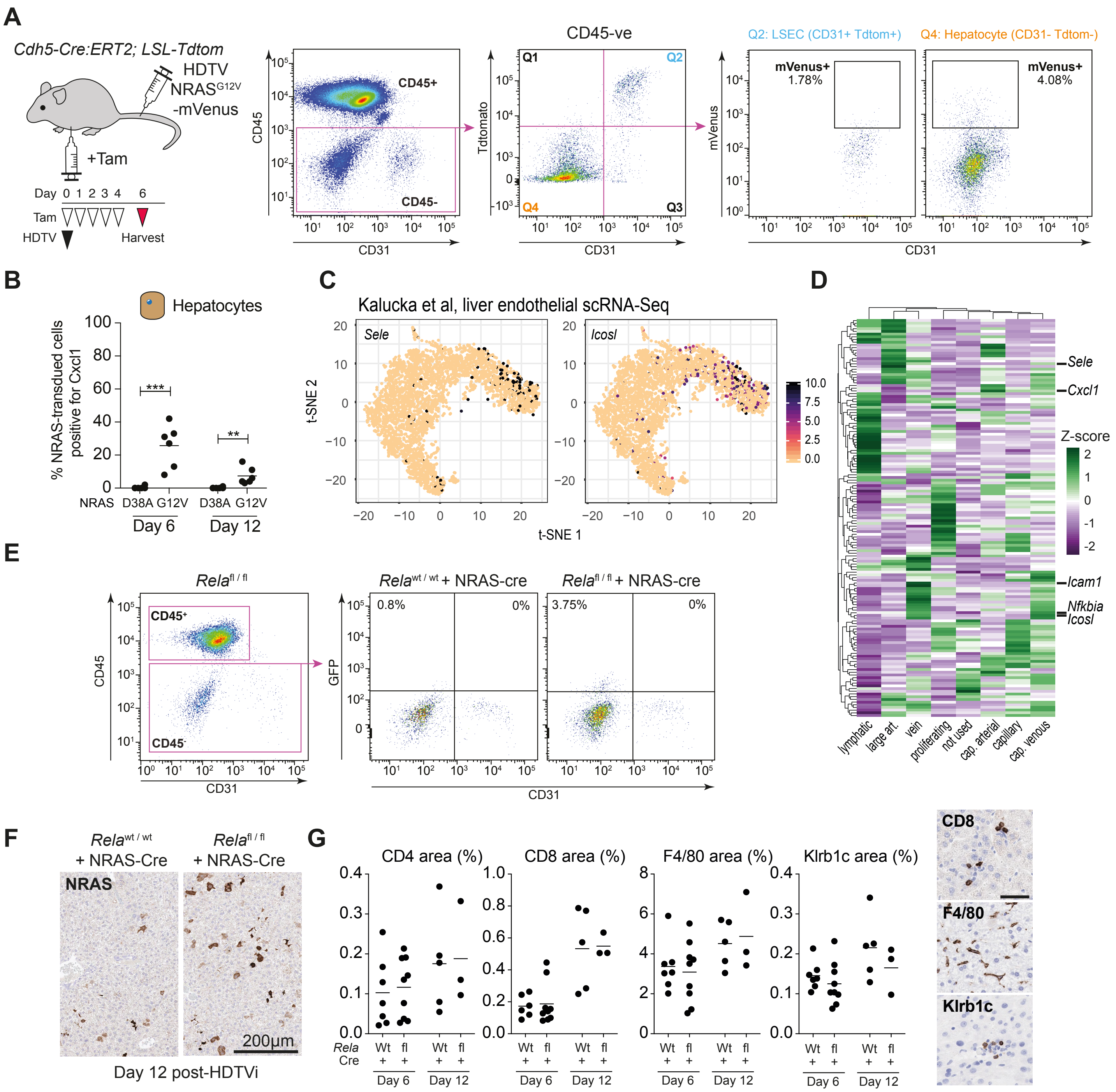

Supplementary figure S3. Senescent hepatocytes induce endothelial NF-κB signalling *in vivo*. (A) Experimental setup: Cdh5-Cre:ERT2; LSL-*TdTomato* endothelial-reporter mice underwent HDTVi with transposons containing oncogenic NRAS^{G12V}-mVenus or non-functional NRAS$^{G12V / D38A}$-mVenus and then intraperitoneal injection with tamoxifen (Tam) leading to TdTomato expression in endothelial cells. After harvesting and digestion, flow cytometry was used to identify the live non-immune cell population (CD45-ve) before analysis of co-expression of CD31 (endothelial cell marker) and TdTomato. Transposon-mediated mVenus expression was enriched in the CD31-TdTomato- hepatocyte population and not in the LSECs. (B) Same experiment as Fig 3A-C; quantification of Cxcl1-expressing mVenus+ hepatocytes at the indicated time points; dots are individual mice; bars are means; data analysed by unpaired Student's t-test; $^{**}P \leq 0.01$,$^{***}P \leq 0.001$. (C) Re-analysis of murine hepatic endothelial scRNA-Seq data from Kalucka et al[29], showing expression of NF-κB targets in similar cell clusters, by tSNE plot. (D) Heatmap of Z-score normalised gene expression in distinct hepatic endothelial subsets, showing expression of NF-κB target genes in veins and venous capillaries. (E) Upon Cre-mediated recombination in Rela$^{fl / fl}$ mice, part of the *Rela* CDS is deleted and a GFP cassette is approximated to the Rela promoter, leading to GFP expression. Upon HDTVi of NRAS^{G12V}-Cre, GFP was only seen in the CD45- CD31- hepatocyte population by flow cytometry analysis. (F,G) Same experimental setup as Fig 3F-J; (F) representative photomicrographs of RAS immunochemistry from indicated conditions (scale bar 200µm). (G) Quantification of CD4, CD8, F4/80 and Klrb1c-positive area from immunohistochemsitry in the indicated conditions; dots are individual mice; bars are means. Example photomicrographs of indicated staining of liver sections by immunohistochemistry; scale bar 50µm.
