# Supplementary material for: Senescence-induced endothelial phenotypes underpin immune-mediated senescence surveillance
Source: Genes Dev. 2022 May 1;36(9-10):533–49. doi: 10.1101/gad.349585.122 (PMC9186388; doi:10.1101/gad.349585.122)
Supplement: Supplemental Material [file supp_gad.349585.122_Supp_FigureS4.ps]

GENESDEV/2022/349585, Supplementary Figure S4, Yin et al.

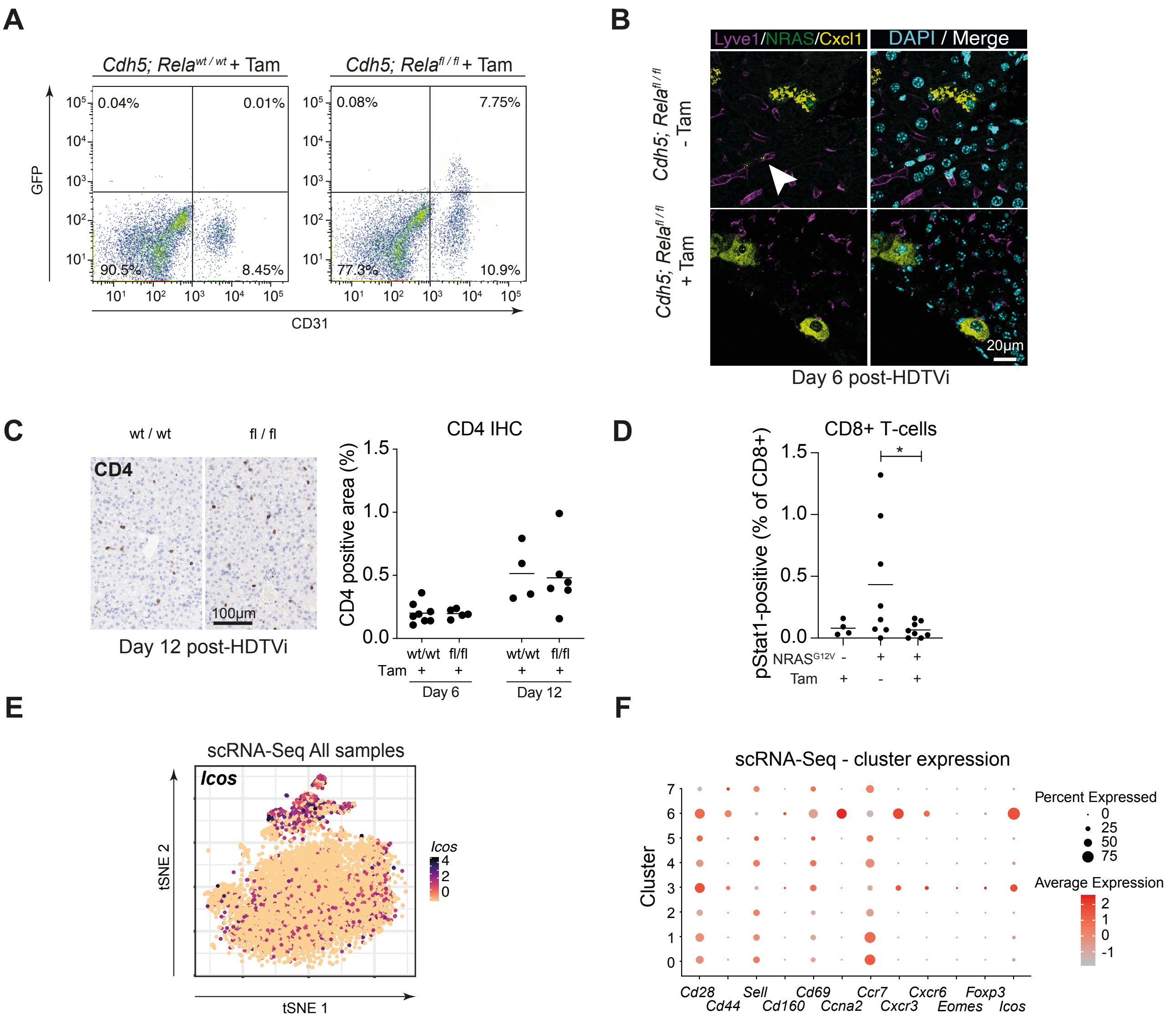

Supplementary figure S4. Non-autonomous induction of endothelial NF-κB signalling regulates senescence surveillance and specific immunocyte recruitment. (A) Same experimental setup as in Fig 4A-B; flow cytometry of live CD45- liver cells demonstrating GFP expression restricted to CD31+ endothelial cells after tamoxifen induction. (B) Representative immunofluorescence of liver sections for the indicated proteins from Cdh5-Cre:ERT2; Rela$^{fl/fl}$ mice treated with or without tamoxifen (Tam) and then harvested 6 days after HDTVi of NRAS^{G12V}; scale bar 20µm. (C) Representative liver photomicrographs of CD4 immunochemistry from indicated conditions (scale bar 100µm) with quantification of CD4+ area; dots are individual mice; bars are means. (D) Flow cytometric analysis of Stat1 phosphorylation at Ser727 in intrahepatic CD8+ T-lymphocytes in the indicated conditions; dots are individual mice; bars are means; data analysed by 1-way ANOVA with Sidak's multiple comparisons test; * $P \leq 0.05$. (E) tSNE clustering of 8152 CD4+ T-cells from 8 mice (same setup as Fig 4F-H), demonstrating $\log_2$ normalised Icos expression. (F) Expression plot demonstrating cluster-specific expression of indicated markers of lymphocyte differentiation and functionality in the indicated CD4+ T-cell clusters from scRNA-Seq data (clusters in Fig 4G).
