# Supplementary material for: Senescence-induced endothelial phenotypes underpin immune-mediated senescence surveillance
Source: Genes Dev. 2022 May 1;36(9-10):533–49. doi: 10.1101/gad.349585.122 (PMC9186388; doi:10.1101/gad.349585.122)
Supplement: Supplemental Material [file supp_gad.349585.122_Supp_FigureS5.ps]

GENESDEV/2022/349585, Supplementary Figure S5, Yin et al.

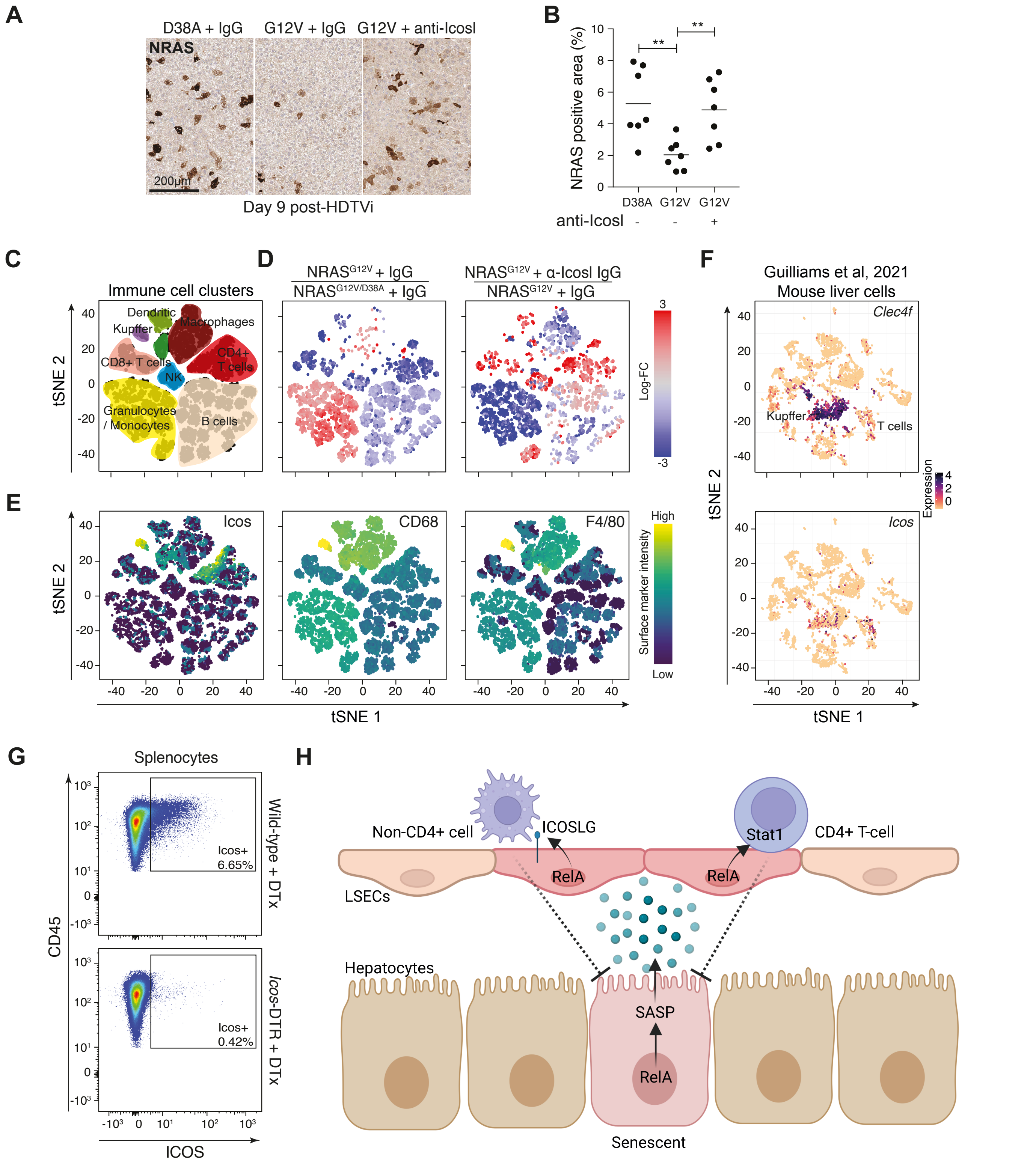

Supplementary figure S5. Icos-Icosl signalling regulates senescence surveillance. (A) wild-type mice injected with NRAS^{G12V}-containing transposons then received intermittent intraperitoneal injection of anti-Icosl or matched IgG control on the indicated days before harvest on day 9 post HDTVi. Representative photomicrographs of RAS immunochemistry from indicated conditions (scale bar 200µm) with quantification (B); dots are individual mice; bars are means; data analysed by 1-way ANOVA with Sidak's multiple comparisons test; $^{**}P \leq 0.01$. (C) t-SNE plots of multiplexed intrahepatic immune cell mass-cytometry (CyToF) data from the same mice in (B) (n = 7 per condition) using metal-tagged antibodies against immunocyte surface markers (See supplementary table). (C-E) Hyperspheres of immune cell phenotypes were identified by typical surface marker expression (C) and coloured according to log-fold change in abundance (D) between NRAS$^{G12V/D38A}$ vs NRAS^{G12V} and NRAS^{G12V} vs NRAS^{G12V}+anti-Icosl conditions. (E) t-SNE plots showing hyperspheres of immune cell phenotypes, coloured by median intensity of Icos, CD68 and F4/80 expression. The colour range is bounded by the 1st and 99th percentiles of the intensities across all cells. (F) Re-analysis of murine hepatic scRNA-Seq data from Guilliams et al[34], showing expression of *Icos* in T-cells and *Clec4f*-positive Kupffer cells, by t-SNE plot. (G) Flow cytometry analysis of efficiency of depletion of Icos+ CD45+ cells after diphtheria toxin administration to wild-type and Icos-DTR mice. (H) Putative model with senescent hepatocytes developing a RELA-dependent SASP that activates RELA in local endothelial cells. This endothelial RELA activation is crucial for modulation of Stat1+ CD4+ T-lymphocytes, upregulation of ICOSLG and senescence surveillance. Interruption of endothelial RELA or ICOSLG expression prevents immune-mediated senescence surveillance.
